# Inactive but Essential: The Role of the Inactive State of E49 in the Mechanism of the Alpha Subunit of Tryptophan Synthase and Its Stand-Alone Blueprint *Zm*BX1

**DOI:** 10.1021/acscatal.5c08026

**Published:** 2026-02-04

**Authors:** Cristina Duran, Sílvia Osuna

**Affiliations:** † Institut de Química Computacional i Catàlisi and Departament de Química, Universitat de Girona, c/Maria Aurèlia Capmany 69, 17003 Girona, Spain; ‡ ICREA, Pg. Lluís Companys 23, 08010 Barcelona, Spain

**Keywords:** tryptophan synthase, stand-alone activity, Molecular Dynamics (MD) simulations, DFT calculations, reaction mechanism

## Abstract

The stand-alone version of the alpha subunit of tryptophan synthase (TrpA), *Zm*BX1, catalyzes the retro-aldol cleavage of indole-3-glycerol phosphate (IGP) at a catalytic efficiency that is approximately 144,000 times higher than that of isolated *Zm*TrpA. Available X-ray crystal structures of *Zm*BX1 and several TrpAs revealed identical overall structures as well as active site geometries, showing high flexibility of the catalytic E49 in both cases. Based on the crystallographic data, E49 was found to adopt an active state in which the carboxylate group is close to IGP for promoting the retro-aldol cleavage as well as an additional inactive state whose catalytic function was unclear. In this work, by using a combination of Molecular Dynamics (MD) simulations and cluster model DFT calculations, we rationalize the effect of the active/inactive conformation of the catalytic E49, as well as how L2 containing the other catalytically relevant residue D60 affects catalysis. The higher levels of retro-aldol activity observed for *Zm*BX1 are attributed to its dual ability to adopt not only active states of the catalytic E49 crucial for retro-aldol cleavage but also inactive states that position E49 in a noncatalytic orientation for disfavoring the reverse aldol reaction back to IGP after product formation. Our combined MD and QM studies elucidate the mechanistic interplay between conformational changes and catalytic steps in *Zm*BX1 and TrpA enzymes. This study highlights the importance of optimizing the conformational changes and chemical steps along the catalytic itinerary for altering and/or improving enzymatic function.

## Graphical abstract

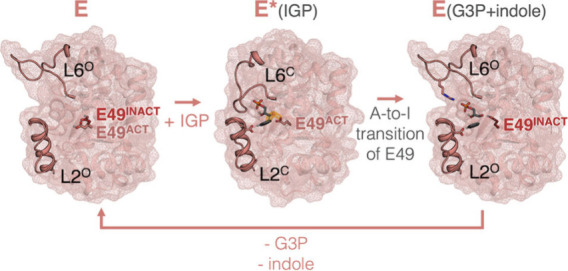

## Introduction

Tryptophan synthase (TrpS) is a heterodimeric complex composed of two α- and two β-subunits (TrpA, TrpB), which is part of the primary metabolism.^1,2^ TrpA catalyzes the retro-aldol cleavage of indole glycerol phosphate (IGP) to yield d-glyceraldehyde 3-phosphate (G3P) and indole, which is tunneled to the TrpB subunit for its condensation with l-serine for L-tryptophan synthesis.^2,3^ The allostery,^4,5^ i.e., the long-range communication, existing between the α- and β-subunits in the αββα heterodimeric TrpS complex renders both TrpA and TrpB subunits highly inefficient in isolation.^3,6^ Such inefficiency in isolation is associated with their restricted conformational flexibility and inability to reach the catalytically productive closed conformations of both TrpA and TrpB for catalysis.^4,7,8^ This contrasts with a paralogue of TrpA from the secondary metabolism of maize, *Zea mays* BX1, which catalyzes the same retro-aldol reaction in the absence of any additional binding partner.^9^ TrpA and *Zm*BX1 are structurally very similar and share the same catalytic Asp and Glu residues (D60/61 and E49/50 for *Zm*BX1/*Zm*TrpA, Figure 1).^10^ Still *Zm*BX1 is 31 times faster than *Zm*TrpA in complex with *Zm*TrpB and 144,000 times more active than the isolated *Zm*TrpA (in *k*_*cat*_/*K*_*M*_).^11^

**Figure 1:**
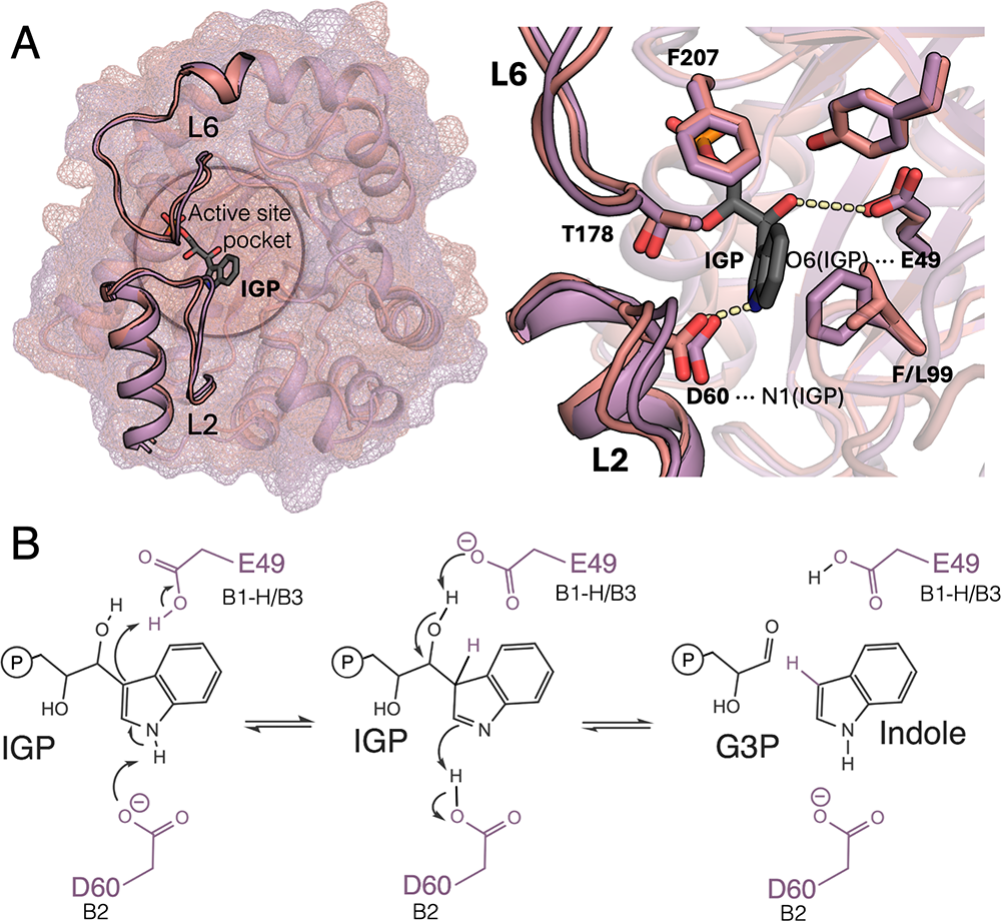
***Zm*** **BX1 catalyzes IGP retro-aldol cleavage at a higher catalytic rate than isolated** ***Zm*** **TrpA despite sharing a high structural similarity**. **A**. Overlay of the structurally similar *Zm*BX1 (shown in pink) and *Zm*TrpA (in purple). Despite the structural similarities, their stand-alone activity differs quite substantially: *Zm*BX1 is 31 times faster than *Zm*TrpA in complex with *Zm*TrpB and 144,000 times more active than the isolated *Zm*TrpA (in terms of *k*_*cat*_/*K*_*M*_).^11^ Zoom of the active site showing the substrate indole glycerol phosphate (IGP), the catalytic E49 (*Zm*BX1 numbering), D60 contained in L2, and active site residues F207, Y170, F/L99, and T178 contained in L6 are represented as sticks. **B**. Postulated retro-aldol reaction of IGP for indole and d-glyceraldehyde 3-phosphate (G3P) formation. E49 has been suggested to act as a proton donor (B1–H) and as the base (B3), whereas D60 catalyzes indolenine tautomerization by abstracting the hydrogen of N1 of IGP.

Kinetic studies of TrpA in complex with TrpB indicated that the chemical step is not rate-determining; instead, it is a conformational change required to reach a catalytically activated E*^IGP^ state.^9,12^ When the aminoacrylate E­(A-A) intermediate is formed at TrpB in the presence of l-serine, the conformational transition of TrpA from the catalytically inactive (E^IGP^) to the activated conformation (E*^IGP^) is promoted.^9,13^ This enhances the rate toward the retro-aldol IGP cleavage 150-fold.^12^ Based on these observations, it was hypothesized that the higher catalytic activity of *Zm*BX1 is due to its superior ability in accessing the catalytically activated E*^IGP^ state.^9^ Recently, we computationally reconstructed the free energy landscape of *Zm*BX1 and compared it to *Zm*TrpA in isolation and in complex.^7^ Our simulations indicated that the catalytically activated E*^IGP^ state corresponds to a conformation in which two active site loops adopt a closed conformation, i.e., loop 6 (L6) and loop 2 (L2) that contains the catalytic D60/61 residue (Figure 1A). The comparison of the conformational landscapes obtained for *Zm*BX1, *Zm*TrpA, and a previously reported *Zm*TrpA variant with L6 implanted from *Zm*BX1 (*Zm*TrpA^L6BX1^)^11^ showed that high levels of stand-alone activity are correlated with a better stabilization of the fully closed (C) E*^IGP^(L6^C^L2^C^) state.^7^ The simulations also indicated that *Zm*BX1 can adopt open states of L2, which trigger the opening of L6 facilitating substrate binding and product release. The synchronized dynamics of L6/L2 is completely missing in *Zm*TrpA, which as a result of the evolutionary constraint to retain indole for its transfer to TrpB cannot efficiently adopt open states of L6. In contrast, in the absence of *Zm*TrpB, L2 containing the catalytic D60/61 is highly disordered and explores wide-open states suggested to be detrimental for catalysis. Conformationally relevant mutations identified with our correlation-based SPM tool developed for enzyme design^7,14^ combined with implantation of L6 of *Zm*BX1 favored the stabilization of the catalytically activated E*^IGP^(L6^C^L2^C^) state, thus enhancing the stand-alone TrpA activity 163-fold (in terms of *k*_*cat*_/*K*_*M*_).^7^

The retro-aldol reaction of IGP was suggested to occur via a push–pull general acid–based mechanism promoted by the catalytic E49 and D60 (in *Zm*BX1 numbering).^9,10^ The importance of these two residues was demonstrated by site-directed mutagenesis: the single conservative mutation E49D,^10^ as well as the single mutants replacing D60 by Asn, Ala, or Tyr result in complete loss of TrpA activity.^15^ L2 contains the catalytic D60 that is suggested to catalyze indolenine tautomerization by abstracting the hydrogen of N1 (Figure 1B).^9^ The role of E49 was more debated.^9^ The analysis of X-ray structures with different derivatives of IGP revealed two possible orientations of E49: an inactive conformation establishing a hydrogen bond with the adjacent Y173 or an active orientation forming a hydrogen bond with the 3′ hydroxyl of IGP (Figure S1).^9,16,17^ However, the X-ray structure of the inactive D60N variant showed that the active orientation of E49 is the one adopted in the presence of the true IGP substrate (PDB: 1A5B, Figure S2A).^18^ In this active conformation E49 can act as the base B3 for abstracting the 3′ hydroxyl proton of IGP. The role of E49 as proton donor (B1–H) at the indole C3 position was also debated.^9^ E49 was adopting the inactive conformation in some X-ray structures with putative tetrahedral transition state analogues bound.^9,16^ However, in one of the reported X-ray structures of TrpA with indole-3-acetylglycine bound (IAG, PDB: 1K7E, Figure S2B), E49 establishes a hydrogen bond with the acetyl oxygen of IAG, thus indicating that E49 is protonated under physiological conditions.^17^ This was also recently supported by a low-pH room temperature X-ray structure (PDB: 8EYS) suggesting E49 to be protonated and mostly in the active state.^19^ Altogether these crystallographic studies suggest that E49 can act as B1–H and B3 of the reaction (Figure 1B); however, the role of the inactive state of E49 in the presence of several IGP analogues is not known. Based on these considerations, two possible mechanisms were suggested: a concerted and a stepwise retro-aldol cleavage. In the stepwise mechanism, E49 (B1–H) protonates carbon C3 of the indole, thus yielding a charged intermediate. This charged intermediate is stabilized by D60, which could potentially abstract the N1 hydrogen of the indole for promoting the indolenine tautomerization.^1,18^ E49 then acts as B3 and abstracts the hydroxyl proton from the formed intermediate, thus promoting C–C bond cleavage for G3P and indole formation. The concerted mechanism occurs in a one-step without formation of any intermediate, which was suggested to be consistent with the hydrophobic microenvironment of the active site pocket for catalysis.^1^ QM/MM studies exploring the TrpA reaction proposed a two-step mechanism for the reaction as a stable zwitterionic intermediate is formed, being the transition state for the protonation of IGP the one with the largest activation energy.^20^ However, in their predicted mechanism, a water molecule bridging the inactive conformation of E49 and IGP was included. In the starting X-ray structure used (PDB: 3PR2, Figure S2C), TrpA presents L6 and L2 closed, but E49 is in the inactive conformation pointing away from the active site. The effect of D60 on the catalysis was not directly assessed. The few available X-ray structures of *Zm*BX1 present the catalytic E49 in the inactive conformation, both in the apo and sulfate-bound states.^9^ The comparison of the inactive state of E49 of *Zm*BX1 with available TrpA structures shows a different inactive orientation, differing in the χ_1_ dihedral of E49 (280° versus ca. 210° in *Zm*BX1 and TrpAs, respectively, Figure S1A). The alternative inactive state observed in some TrpAs (that has the same χ_1_ but differs in χ_2_, Figure S2B) is due to E49 interaction with Y173, which in *Zm*BX1 and *Zm*TrpA is instead a phenylalanine (F168 and F173, respectively).

In this study we aim to rationalize the catalytic mechanism of *Zm*BX1, which displays substantially higher levels of retro-aldol activity as compared to its stand-alone blueprint TrpA.^7,9,11^ We study the retro-aldol mechanism in the absence of any additional water molecule in the catalytically activated E*^IGP^ state. By using a combination of Molecular Dynamics (MD) simulations, cluster model DFT calculations, and hybrid Quantum Mechanics/Molecular mechanics (QM/MM), we rationalize the effect of the active/inactive conformation of the catalytic E49, as well as how the interaction of D60 contained in L2 with IGP affects catalysis. Our study indicates that a stepwise mechanism is followed, favoring the formation of a charged intermediate over the neutral intermediate after indolenine tautomerization. The higher levels of retro-aldol cleavage observed for *Zm*BX1 activity are attributed to its dual ability to adopt active states of the catalytic E49 crucial for retro-aldol cleavage but also inactive states that contribute to product stabilization, thus disfavoring the reverse aldol reaction back to IGP formation.

## Results

### *Zm*BX1 Adopts Active and Inactive States of E49 Even in the Presence of IGP

The combined analysis of available X-ray structures of *Zm*BX1 and several TrpAs reveals a lack of preorganization, i.e., a large flexibility, of E49 adopting either the catalytically active state (with χ_1_ of ca. 185°, Figure S1B) or two different catalytically nonoptimal inactive conformations (both presenting a χ_1_ of ca. 280°). To characterize and evaluate the conformational flexibility of E49 in *Zm*TrpA and *Zm*BX1, we conducted multiple replica unbiased nanosecond time scale MD simulations in the presence of IGP (see Methods). The reconstructed conformational landscapes represented in Figure 2 are based on the dihedral χ_1_ of the catalytic E49 (*x*-axis) and the catalytic distance between the carboxylate carbon of E49 and the 3′ hydroxyl of IGP (*y*-axis). For comparison, the crystallographic values obtained for the IGP-bound structure of the D60N mutant are represented. Although in available *Zm*BX1 crystallographic structures E49 adopts the inactive state (χ_1_ of ca. 280°), the reconstructed conformational landscapes indicate that both active (χ_1_ of ca. 185°) and inactive states (290°) are equally visited at short catalytic distances of less than 4 Å (Figures 2, S3). These simulations indicate that E49 can easily transition between both states in the presence of IGP, thus suggesting that both states could potentially play a role in the reaction (Figure S4). MD simulations performed in the absence of IGP provide the same conclusions: both active and inactive states of E49 are explored, the inactive state being slightly more populated in line with the crystallographic data (Figures S3–S5).

**Figure 2:**
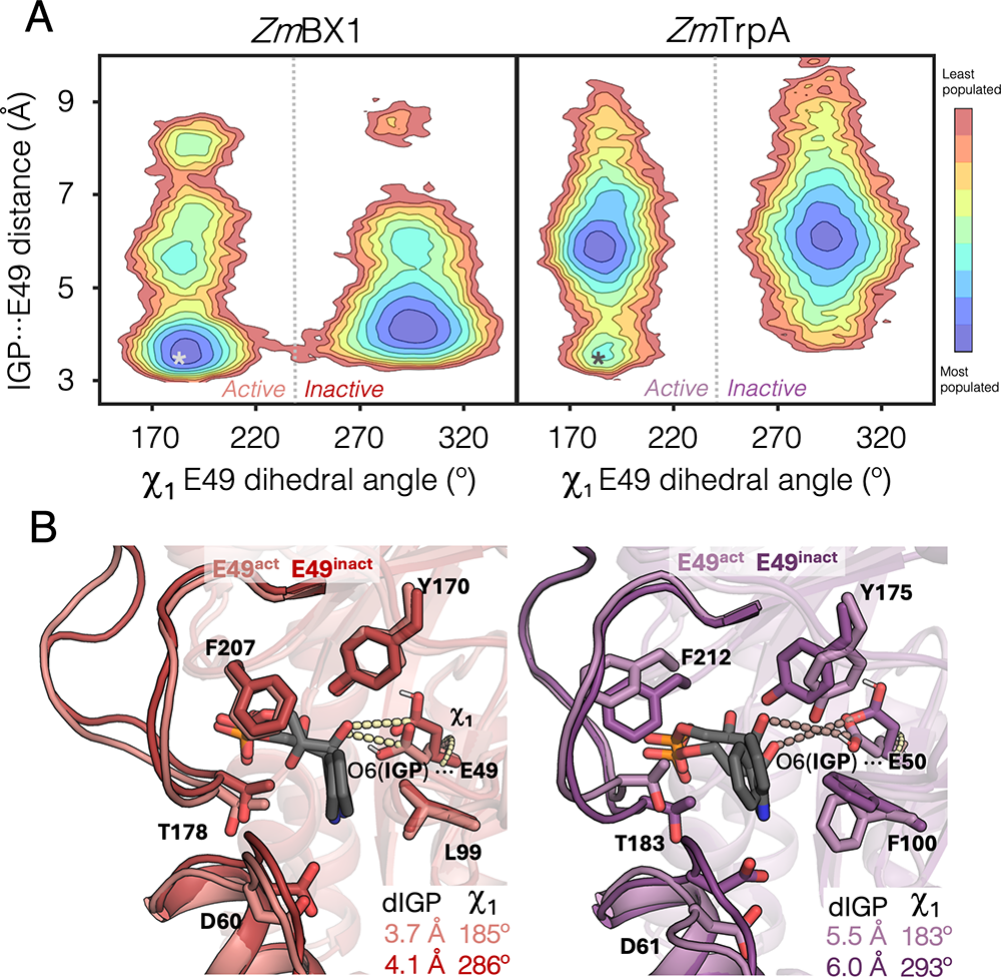
**Conformational landscapes of** ***Zm*** **BX1 and isolated** ***Zm*** **TrpA in the presence of IGP**. **A**. The reconstructed conformational landscapes are based on the dihedral χ_1_ of the catalytic E49 (*x*-axis) and the catalytic distance between the carboxylate carbon of E49 and the 3′ hydroxyl of IGP (*y*-axis). Active states of E49 present χ_1_ of ca. 185°, whereas inactive states present values of 290°. For comparison, the crystallographic values obtained for the IGP-bound structure of the D60N mutant are represented with asterisks. Most stable conformations are colored in blue, whereas the least stable ones are in red. **B**. Overlay of a representative structure of the active and inactive states presented a catalytic distance below 4 Å for *Zm*BX1 (left panel) and ca. 6 Å for *Zm*TrpA (right panel). MD simulations of *Zm*TrpA are performed in the absence of the *Zm*TrpB binding partner.

Active and inactive states of E49 are also observed in the reconstructed conformational landscapes of several *Zm*TrpA systems displaying different levels of stand-alone activity Figures 2A, S3, and S6–S8). However, both active/inactive conformations display dramatically different catalytic E49-IGP distances: whereas short distances (<4 Å) are stabilized in those systems presenting a higher catalytic activity (i.e., *Zm*TrpA in complex with *Zm*TrpB, and the rationally designed *Zm*TrpA^SPM6‑L6BX1^, Table S1), substantially longer distances (ca. 6 Å) are favored for isolated *Zm*TrpA (shown in Figure 2A) and *Zm*TrpA^L6BX1^ that have poor stand-alone catalytic activities (Figure S6).^7,11^ The analysis of the water content of the active site along the MD simulations indicates no water accumulation between IGP and E49, even when E49 adopts the inactive state (Figure S9). This finding therefore does not support a water-mediated C3 protonation of IGP by the inactive state of E49, as reported previously.^20^

These MD simulations indicate that high levels of retro-aldol activity require E49 to adopt both active and inactive states, especially at short catalytic IGP-E49 distances (<4 Å). While the active state is the catalytically relevant conformation for promoting retro-aldol cleavage, the role of the inactive state, which in our MD simulations of *Zm*BX1 is rather stable, is, as mentioned before, not known.

### The Inactive State of E49 Disfavors the Reverse Aldol Reaction in *Zm*BX1

Intrigued by how the different conformations of E49 influence the retro-aldol/aldol reaction, we computationally studied the reaction mechanism considering both active and inactive states of the catalytic E49. Our cluster model and QM/MM calculations, based on the available X-ray structure (PDB: 1TJR), indicate that in the presence of IGP, the active conformation of protonated E49-H is substantially more favored than the inactive state (by ca. 3 kcal/mol, Figure 3, Figure S10). Both active and inactive states can establish a hydrogen bond with the 3′ hydroxyl group of IGP; however, only the active state of E49 properly positions the syn hydrogen toward the C3 carbon of the indole ring for protonation (Figure 4). The first transition state (TS1) corresponds to indole C3 protonation and presents an activation enthalpy of ca. 12.2 kcal/mol (Figures 3, S10 and Table S2). This generates a charged intermediate INT1^D60^ exhibiting a high stability, presumably thanks to the interaction with the other catalytically relevant residue D60 contained in L2 (Figures 1, 3, and S10). It was postulated that D60 could potentially abstract the N1 hydrogen of the indole for promoting the indolenine tautomerization.^1,9,18^ In the optimized charged intermediate, a slightly longer hydrogen bond distance of 1.07 Å between the indole nitrogen and hydrogen is observed at INT1^D60^ (1.02 Å at the RC). Our calculations indicate that N1 hydrogen abstraction by D60 after C3 protonation generating a neutral intermediate INT1^D60‑H^ is also possible, and in fact, both charged INT1^D60^ and neutral INT1^D60‑H^ intermediates present similar relative stabilities of ca. 0.8 kcal/mol. The transition state for N1 (de)­protonation TS^PROT^ is 1.1 kcal/mol (i.e., a barrier of ca. 0.3 kcal/mol from INT1^D60^), thus indicating that both INT1/INT1^D60‑H^ intermediates exist and are equally favored (Figures 3, S11). We also evaluated the possibility of a concerted TS1^con^ in which D60 abstracts N1 hydrogen of the indole moiety, while E49-H protonates the C3 position of IGP; however, all attempts to locate this transition state yielded the stepwise TS1 (Figure S12). We additionally located the C3 protonation TS1 considering D60 protonated (TS1^D60‑H^), but the activation enthalpy is higher (ca.7.8 kcal/mol higher than TS1, Figures 3, S12).

**Figure 3:**
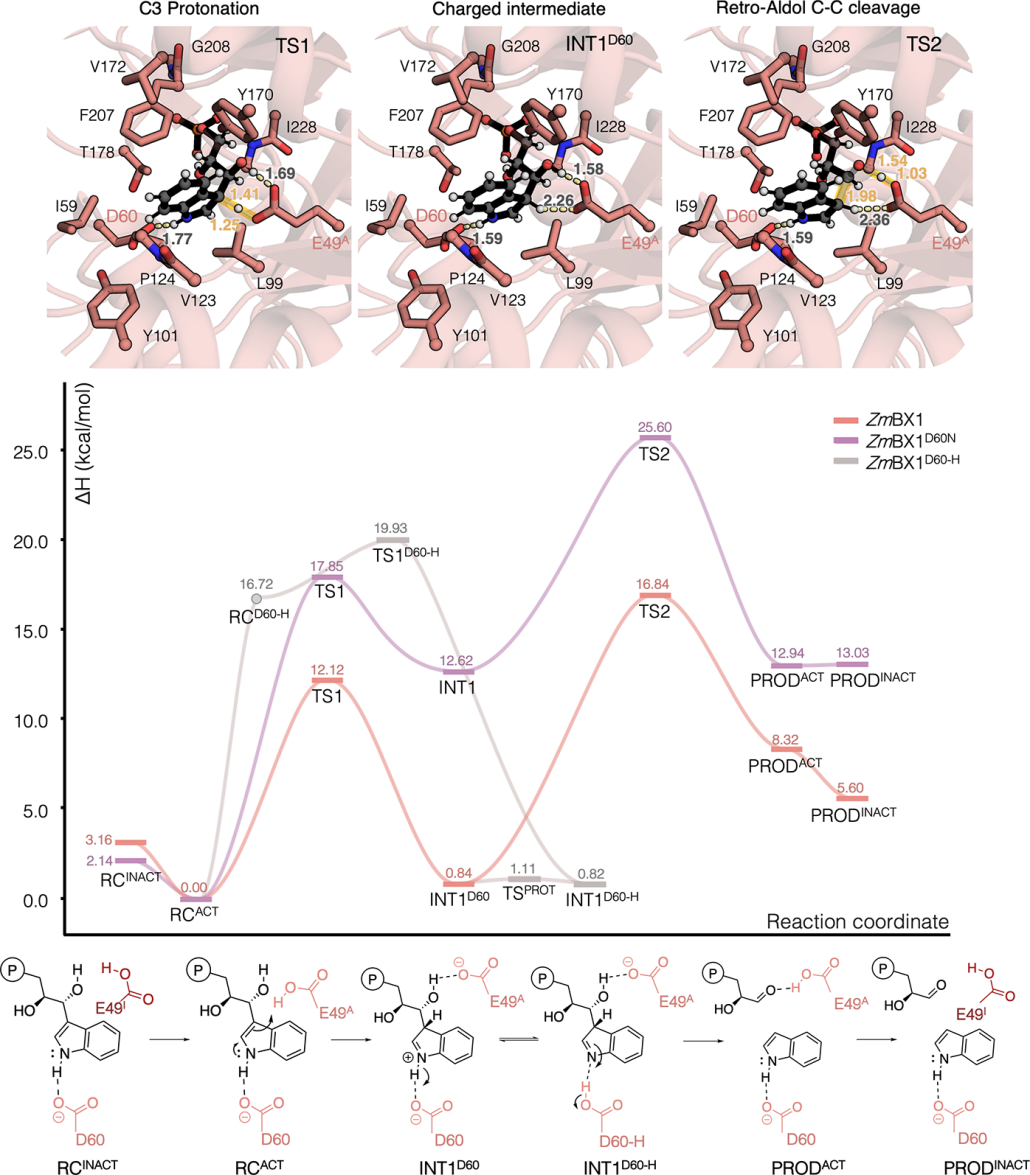
**DFT optimized reaction mechanism of E49 and D60 catalyzed retro-aldol cleavage in TrpA/*Zm*BX1 and D60N variant**. The computed reaction profile for the wild-type TrpA/*Zm*BX1 enzymes is shown in pink, whereas the mechanism obtained for the D60N variant is shown in purple. The reaction profile is obtained using a cluster model composed by residues: E49, I59, D60, L99, Y101, V123, P124, Y170, V172, T178, F207, G208, I228, G229. DFT optimized structures of TS1, INT1^D60^, and TS2 are included with the most relevant distances in Å. IGP is shown as gray spheres and black sticks. The atoms kept frozen during the optimization are marked with a pink sphere. The reaction profile of the additional stepwise TS1 yielding the neutral INT1^D60‑H^ intermediate is also included (the RC with IGP deprotonated and D60-H is not a stable minimum, marked with a gray dot). All optimized structures are displayed in Figures S11–S16. For TrpA/*Zm*BX1, QM/MM calculations were additionally carried out (Figure S10 and Tables S2–S3), confirming the conclusions obtained from the cluster model calculations. The obtained barrier for the rate-determining retro-aldol C–C bond cleavage as well as the enthalpy of the E·S complex is in line with the previously reported experimental values.^21^

**Figure 4:**
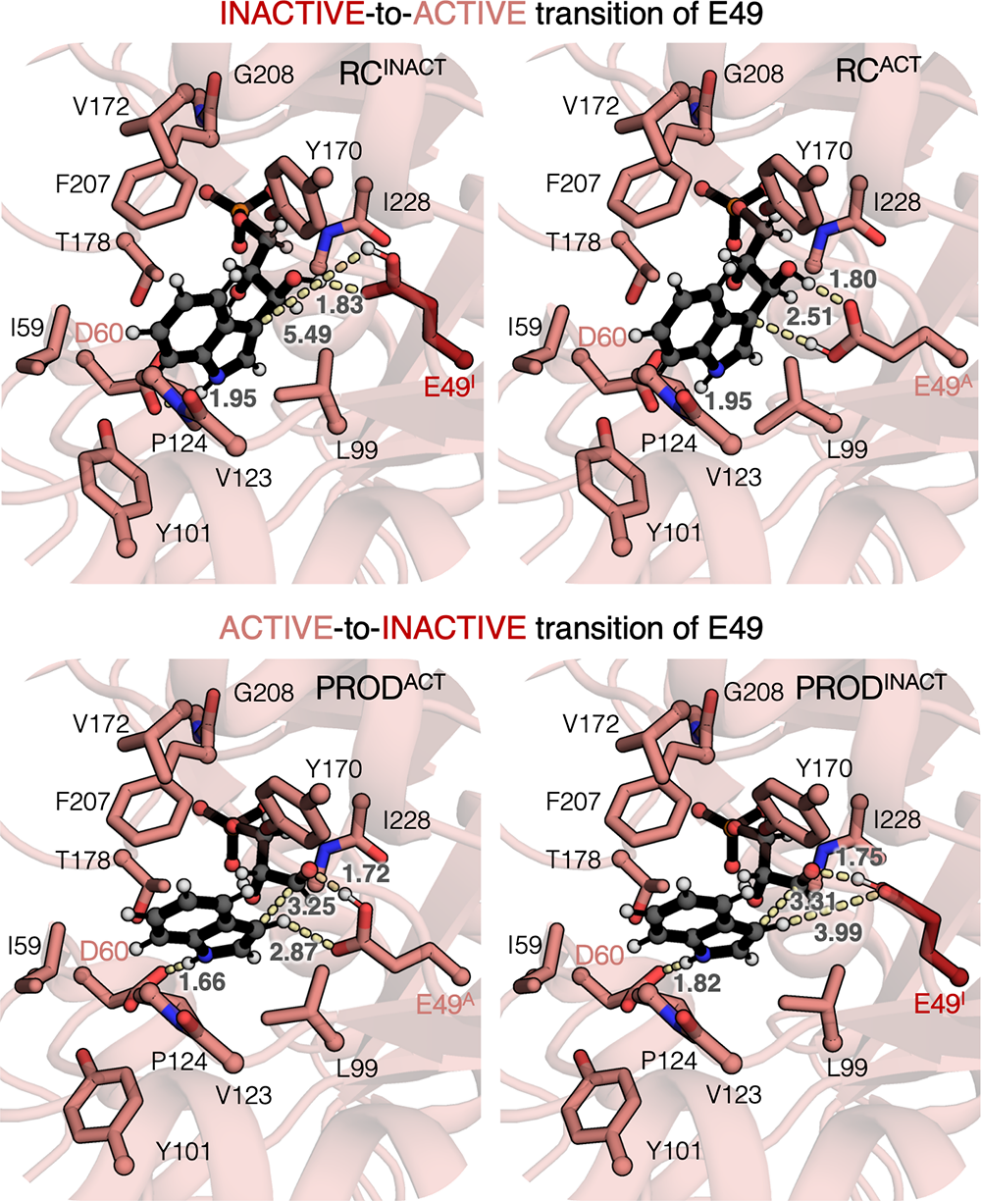
**Active to inactive transition of E49 at the reactant and product complexes**. The most relevant distances are shown in Å. IGP is shown as gray spheres and black sticks. The atoms kept frozen during the optimization are marked with a pink sphere. When E49 adopts the active state, is colored in pink (E49^A^), whereas dark brown is used to represent the inactive conformation (E49^I^).

The next step involves deprotonation of the 3′ hydroxyl group of IGP by E49 at INT1^D60^, which triggers the C–C bond breaking for generating indole and G3P. The associated TS2 presents an activation enthalpy of ca. 16.8 kcal/mol and, as shown by both QM/MM and cluster model calculations (Table S3), corresponds to the rate-limiting step of the overall reaction mechanism. This activation enthalpy is in line with the reported rate constant for *Zm*BX1 of 2.8 s^–1^.^7^ All attempts to locate the retro-aldol TS from the neutral INT1^D60‑H^ intermediate led to the same TS2 with indole N1 protonated and D60 deprotonated.

In line with the known reversibility of the reaction and previous thermodynamic investigations of the reactions catalyzed by tryptophan synthase,^21^ formation of E·P (G3P + indole) is slightly endoergonic. The experimentally reported reaction enthalpies toward IGP synthesis are ca. −10 kcal/mol.^21^ Interestingly, our calculations indicate that the stability of the E·P complex depends on the conformation of E49: in the active state when indole and G3P are present in the active site, a relative enthalpy of +8.3 kcal/mol is obtained (as compared to RC), whereas in the inactive state, the products are stabilized ca. 3 kcal/mol (the E·P complex is +5.6 kcal/mol). This stabilization is mostly attributed to the different orientations of the side chain of L99 and E49 (Figures S13 and S14). When E49 is in the inactive conformation, an increase in the distance between indole and G3P is observed, but also E49 is not well positioned for protonating the carbonyl oxygen of G3P. Both features are needed for the backward formation of INT1 (Figures 4 and S10, S13–S14). The inactive-to-active transition of E49 has a small barrier, as shown with the MD simulations (Figures S4, S7, and S15). These findings suggest that whereas the active state of E49 is the catalytically relevant conformation, the inactive state positions the carboxylate group far from G3P and indole, which stabilizes G3P and indole in the active site and hampers the reverse aldol reaction back to IGP formation.

### D60 Stabilization of the Charged Intermediate Is Crucial for Catalysis

The elucidated stepwise retro-aldol mechanism indicates the formation of a charged intermediate INT1^D60^, which is stabilized via hydrogen bonding with the catalytic residue D60 contained in flexible L2. As postulated in previous studies, we find that D60 can deprotonate the hydrogen of N1 of indole for favoring indolenine tautomerization, thus yielding neutral intermediate INT1^D60‑H^ being equally stable as INT1^D60^. As described above, mutation of D60 to either Asn, Ala, or Tyr depletes completely TrpA catalytic activity.^15^ The available X-ray structure of the D60N mutant in the presence of IGP reveals that aside from the mutation and E49 adopting the active conformation, an identical active site pocket to the wild-type enzyme is obtained.^18^ Based on this X-ray structure, we computationally explored the effect of the D60N mutation in the reaction profile. As shown in Figure 3, D60N dramatically affects the stability of the E·P (G3P + indole) complex, which is ca. 13 kcal/mol higher in enthalpy than RC. As initially postulated, the absence of the carboxylate group of D60 profoundly affects the stabilization of the neutral intermediate INT1^D60^, which lies ca. 13 kcal/mol higher in energy than RC. As observed for *Zm*BX1, the retro-aldol C–C bond cleavage (TS2) is the rate-determining transition state; however, a much larger activation enthalpy of more than 25 kcal/mol is obtained (as opposed to the 16.8 kcal/mol for *Zm*BX1). In the mutant, the E49 conformation has a minor effect on the stabilization of the generated products (indole + G3P), as in both active/inactive states a relative enthalpy of ca. 13 kcal/mol is obtained. MD simulations performed for the D60N variant indicate a rather long catalytic distance between IGP and E49 (distances below 4.5 Å are hardly sampled, Figure S17). Still, E49 can adopt both active and inactive states, as observed for the other systems (Figures S18 and S19).

Our calculations are therefore in line with the experimental findings of a large impact of D60N mutation in TrpA catalytic activity.^15^

### L2 Dynamics Impact E49 Deactivation

The interaction between D60 and the nitrogen of indole of IGP has been shown to be crucial for stabilizing the generated intermediates along the reaction and promoting the retro-aldol cleavage. Considering that D60 is contained in L2, such an interaction is therefore dependent on the L2 conformation. As observed in X-ray structures, E49 can adopt active and inactive states, which we found to be crucial both for catalyzing the retro-aldol reaction and for disfavoring the reverse reaction back to the starting IGP substrate. We hypothesized that the L2 opening, which clearly hampers D60-IGP interaction, could potentially stabilize the inactive state of E49 for disfavoring the aldol reaction and favoring the release of the generated G3P and indole products. To that end, we evaluated how the L2 closed-to-open conformational change affects E49 active/inactive states (Figure 5). Interestingly, in *Zm*BX1, we observed that active and inactive conformations of E49 are sampled independently of L2 conformations, i.e., loop 2 conformation does not influence the E49 active/inactive state. However, the inactive state is slightly more stable at both open and closed conformations of L2 (Figures 5B, S20). Despite the active and inactive states of E49 presenting different χ_1_ dihedrals in *Zm*BX1, both states position one of the two carboxylate oxygens at the exact same position (Figure 5B). However, the active state of E49 presents a much shorter catalytic distance, as it positions the other carboxylate oxygen at less than 3 Å.

**Figure 5:**
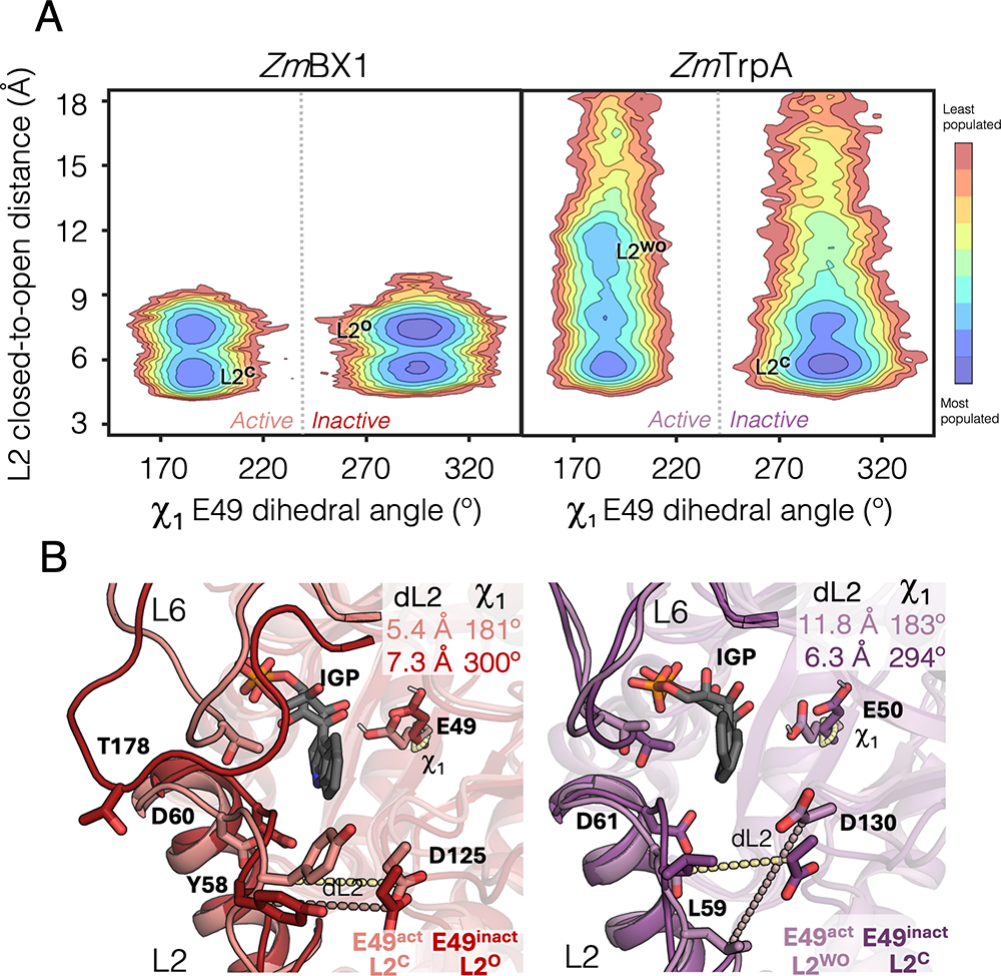
**Conformational landscapes of** ***Zm*** **BX1 and isolated** ***Zm*** **TrpA in the presence of IGP**. **A**. The reconstructed conformational landscapes are based on the dihedral χ_1_ of the catalytic E49 (*x*-axis) and the L2 closed-to-open distances computed considering the distance between the alpha carbon of Y58 and D125 (*y*-axis, in Å). Active states of E49 present χ_1_ of ca. 185°, whereas inactive states present values of 290°. Closed states of L2 (L2^C^) present distances of ca. 5–6 Å, whereas L2 open (L2 °) and widely open (L2^WO^) states present distances of ca. 7 and 12 Å, respectively. Most stable conformations are colored in blue, whereas the least stable ones are colored in red. **B**. Overlay of representative structures of the active and inactive states presenting L2 in either closed or (widely) open conformations for *Zm*BX1 (left panel) and *Zm*TrpA (right panel). MD simulations of *Zm*TrpA are performed in the absence of the *Zm*TrpB binding partner. In complex calculations are shown in Figure S20B.

Isolated *Zm*TrpA and our *Zm*TrpA^SPM4‑L6BX1^ variant that is 890-times less active than *Zm*BX1 (in terms of *k*_*cat*_ */K*_*M*_, Table S1) favor both active and inactive states of E49 but only at L2 closed conformations (Figures 5A, S20). Similar to *Zm*BX1, active and inactive conformations of E49 are sampled independently of L2 conformations also for *Zm*TrpA in complex with *Zm*TrpB (Figure S20B), although the active state of E49 when L2 is open is substantially less stable compared to *Zm*BX1. This observation is also in line with the lower catalytic activity of *Zm*TrpA in complex with *Zm*TrpB as compared to *Zm*BX1.^7,11^

In the absence of IGP, *Zm*BX1 preferentially adopts the open states of L2 with E49 at the active state (Figure S21). It is worth mentioning that for *Zm*BX1 the overlay of active and inactive states of E49 shows a similar conformation of the carboxylate group despite presenting different χ_1_ dihedrals (Figure S21B). This is also observed in the QM/MM optimized structures of the E·P complex of *Zm*BX1 (Figure S10). The inefficient isolated *Zm*TrpA explores the active state of E49 mostly when L2 is open, and the inactive state is favored at the closed conformations of L2. As opposed to *Zm*BX1, active and inactive states of E49 are dramatically different, being the active state the only one relevant for promoting the retro-aldol reaction (Figure S21C). Similar to *Zm*BX1, *Zm*TrpA^SPM4‑L6BX1^ that presents a higher stand-alone catalytic efficiency as compared to *Zm*TrpA favors both active and inactive states of E49 in the absence of IGP when L2 is open. These simulations therefore indicate that the importance of L2 conformation for catalysis is 2-fold: first, it affects the D60-IGP interaction crucial for stabilizing the developed charged intermediates along the mechanism, and second, in the most efficient systems, E49 can adopt the active and inactive states at both closed and open conformations of L2.

## Discussion and Conclusions

The study of the conformational dynamics of *Zm*BX1 and several TrpAs displaying different levels of stand-alone activity has revealed a high flexibility of E49, in line with the previously reported X-ray structures that indicate two possible states for E49.^9,16,17^ These two states of E49 found crystallographically were proposed to correspond to an active state (χ_1_ of ca. 185°) that positions the carboxyl group close to IGP for promoting the reaction and an inactive state (χ_1_ of ca. 290°) with the carboxylate pointing outside the active site pocket. The role of this inactive state of E49, however, was not clear. Our MD simulations indicate a rather high stability of the so-called inactive state of E49, which we decided to evaluate its impact into the catalytic mechanism by means of cluster model calculations and QM/MM calculations. Based on the crystallographic observations, two potential mechanisms were suggested for the retro-aldol cleavage: a concerted and a stepwise mechanism.^1,18^ Previous QM/MM calculations considering a water molecule bridging the inactive state of E49 and IGP suggested a two-step mechanism, being the water-assisted protonation of IGP the step with the largest activation energy.^20^ Our DFT calculations also show that the retro-aldol cleavage takes place in a stepwise manner: first C3 protonation of IGP is accomplished thanks to the active state of the protonated catalytic E49, which with the help of D60 deprotonates the N1 nitrogen of the indole moiety for promoting indolenine tautomerization. In contrast to the Teixeria et al. study,^20^ this direct protonation of IGP from the active state of E49 is not water-mediated and has an activation enthalpy of ca. 12 kcal/mol. In fact, the analysis of the most conserved water molecules in the MD trajectories indicates no water accumulation between the IGP and E49 in any of the analyzed systems. The rate-determining step of the overall reaction corresponds to the carbon–carbon bond cleavage and deprotonation of the 3′-hydroxyl group of IGP by E49. Our cluster model and QM/MM calculations provide very similar energy profiles and indicate that the inactive state of E49 positions the carboxylate group far from the generated G3P product, which favors a longer distance between the two generated products. Both points are essential for disfavoring the backward aldol reaction toward IGP formation. The inactive state of E49 also helps in stabilizing the E·P (G3P + indole) complex.

The key role of D60 in the mechanism is further elucidated by the computed reaction profile for the D60N variant. The lack of the carboxylate group able to stabilize and abstract N1 hydrogen during the course of the reaction has a negative impact on the overall reaction profile both from a kinetic and a thermodynamic point of view. The E·P (G3P + indole) complex is destabilized by ca. 8 kcal/mol. This is in line with the experimental observation of a detrimental effect of this mutation on TrpA catalytic activity.^15^

D60 is contained in flexible L2, which together with L6 needs to be synchronized to adopt the closed conformation for reaching the catalytically activated E*IGP state for catalysis. Our MD simulations indicate that L2 conformation affects D60-IGP interaction, but it also modulates E49 deactivation for disfavoring the backward aldol reaction. Based on our previous publication^7^ and this combined MD, cluster model, and QM/MM study, we postulate the following mechanism for *Zm*BX1 (Figure 6): (1) L6 and L2 openings in the absence of IGP favor the E49 active state, which might favor IGP binding in the active site pocket, (2) IGP binding promotes the formation of the catalytically activated L6 and L2 closed state E*­(IGP), which properly positions the other catalytic residue D60 in place for stabilizing the charged reaction intermediate formed after C3 protonation of IGP by the active state of E49, (3) this active state E49^A^ promotes the retro-aldol cleavage of IGP generating indole and G3P, which require the E49 active-to-inactive transition for disfavoring the reverse aldol formation, and (4) L2 and L6 openings promote product release thanks to R181,^7^ which as described in our previous publication establishes a salt bridge with the phosphate group of IGP/G3P when open states of L6 are visited. This allowed a new reaction cycle to start. The alpha subunit of tryptophan synthase when in complex with TrpB follows the same mechanism; however, some differences are observed: (1) TrpB binding disfavors widely open L6 and L2 states of TrpA and stabilizes the catalytically activated L6 and L2 closed state for catalysis, as described in our previous publication;^7^ (2) it stabilizes the active state of E49 with short catalytic E49-IGP distances. As opposed to that of *Zm*BX1, the inactive state of E49 in *Zm*TrpA:*Zm*TrpB displays large catalytically unproductive E49-IGP distances. Our combined MD and QM-based study also indicates that the low stand-alone catalytic efficiency of isolated *Zm*TrpA is due to (1) longer IGP-E49 catalytic distances, resulting in a poorer preorganization for catalysis, (2) higher flexibility of L2 leading to widely open states detrimental for properly positioning D60 for the reaction to occur, and (3) at both open and closed conformations of L2, restricted ability to adopt active and inactive states of E49, being the latter necessary to prevent the backward aldol reaction. All of these aspects collectively lead to less efficient catalysis. Our study therefore elucidates the intricate link between conformational changes and their effect on the chemical steps on tryptophan synthase A and its stand-alone blueprint *Zm*BX1.

**Figure 6:**
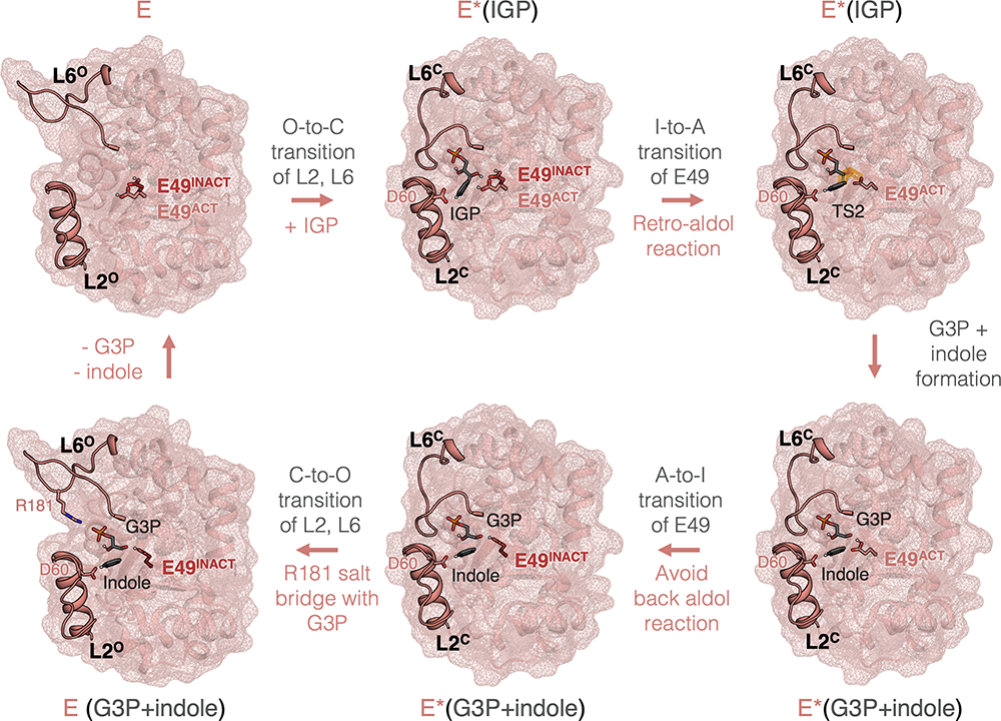
**Schematic representation of the elucidated mechanism of** ***Zm*** **BX1 and the alpha subunit of tryptophan synthase in complex with TrpB**. Conformational changes involve the open-to-closed (O-to-C) transitions of L6 and L2 and the active-to-inactive (A-to-I) transition of E49 (or vice versa). The active (A) state of E49 is crucial for catalyzing the retro-aldol cleavage, whereas the inactive (I) state is crucial for disfavoring the backward aldol reaction. R181 establishes a salt bridge with the phosphate group of IGP/G3P for favoring substrate binding and product release, as described in a previous publication.^7^

## Methods

### Molecular Modeling System Preparation

The starting structures for the seven systems (*Zm*BX1, *Zm*TrpA, *Zm*TrpS, *Zm*TrpA^L6BX1^, *Zm*TrpS^L6BX1^, *Zm*TrpA^SPM4‑L6BX1^, *Zm*TrpS^SPM4‑L6BX1^) were generated with the multimer version of the AlphaFold2 (AF2)^22^ neural network.

The MD parameters for the substrate IGP and the A-A intermediate were generated with the antechamber and parmchk2 modules of AMBER20 ^23^ using the second generation of the general amber force-field (GAFF2).^23,24^ The IGP substrate and A-A intermediate were optimized at the B3LYP/6–31G­(d) level of theory including Grimme’s dispersion correction with Becke-Johnson Damping (D3-BJ) and the polarizable conductor model (PCM) (diethyl ether, ε = 4.2) as an estimation of the dielectric permittivity in the enzyme active site.^25^ The partial charges (RESP model)^26^ were set to fit the electrostatic potential generated at the HF/6–31G­(d) level of theory. The charges were calculated according to the Merz–Singh–Kollman^27^ scheme using the Gaussian16 software package.^28^ The protonation states were predicted using PROPKA.^29^ For *Zm*BX1, *Zm*TrpA, *Zm*TrpA^L6BX1^ , and *Zm*TrpA^SPM4‑L6BX1^, the protonation state of the catalytic residue E49/50 was neutral (i.e., GLH49 and GLH50), as described in the TrpA mechanism. For the heterocomplex simulations, the protonation state of the TrpB catalytic residue K84 was neutral (i.e., LYN84), as is described in the mechanism.^3^ The enzyme structures were solvated in a pre-equilibrated truncated octahedral box of 10 Å edge distance using the OPC water model and neutralized by the addition of explicit counterions (i.e., Na^+^) using the AMBER20 leap module. All MD simulations were performed using a modification of the amber99 force field (ff19SB).^30^

### MD Simulation Details

The MD equilibration phase was done following the protocol described by Roe and Brooks with small differences fine-tuned to our systems (see the SI for more details).^31^ A total of 10 replicas of equilibration and production runs were performed, reaching a total simulation time of 5 μs/system (10 replicas × 500 ns) for *Zm*BX1, *Zm*TrpA, *Zm*TrpA^L6BX1^, *Zm*TrpA^SPM4^, *Zm*TrpA^SPM6^, and *Zm*TrpA^SPM4‑L6BX1^ systems. For the heterocomplexes (i.e., *Zm*TrpS, *Zm*TrpS^L6BX1^, and *Zm*TrpS^SPM4‑L6BX1)^), 6 replicas of equilibration and production runs were performed, reaching a total simulation time of 2.4 μs for each system (6 replicas × 400 ns). The MD trajectories were analyzed using the Python packages MDTraj^32^ and pytraj,^33^ which are part of the cpptraj package,^34^ MDAnalysis,^35^ and PyEMMA.^36^

### Quantum Mechanical (QM) and QM/MM Calculations

The cluster models and QM/MM calculations were performed from the X-ray structure of *Zm*BX1 (PDB code: 1TJR). For the Asp60Asn mutation, the same cluster model was used, incorporating Asn60 in the same conformation of the X-ray structure of this single mutant variant (PDB code: 1A5B).^18^ All the optimization and high-level energy calculations were performed with Gaussian16.^28^ The systems were described with the B3LYP functional with the GD3 dispersion correction^37^ and adding solvation corrections through the Solvation Model based on Density (SMD).^38^ 6–31G­(d) was used as the basis set. All energies were calculated by performing single-point calculations on the optimized geometries using the functional ωB97XD^39^ with the 6–311+G­(2d,2p) basis set.

QM/MM calculations were performed by using the ONIOM method. MolUP was used to prepare the inputs. The QM region comprised residues E49, D60, and Y170 and the substrate (62 atoms and 3 H-link atoms). All residues and water molecules around 10 Å from the QM region constitute the active region. The residues in the MM region are treated with an amber ff14SB force field. A two-step optimization protocol was applied: an initial mechanical embedding optimization followed by electrostatic embedding. The QM region was treated at the B3LYP/6–31G* level for optimizations with single-point energy refinements at the ωB97X-D/6–311+G­(2d,2p) level. Transition states were confirmed by frequency analysis.

All the computational data obtained from the single-point calculations have been uploaded onto the IOCHEM-BD platform (https://doi.org/10.19061/iochem-bd-4-86).

## Supplementary Material



## Notes

Full details of the computational methods and experimental protocols (PDF)
