# Supplementary material for: Inactive but Essential: The Role of the Inactive State of E49 in the Mechanism of the Alpha Subunit of Tryptophan Synthase and Its Stand-Alone Blueprint ZmBX1
Source: ACS Catal. 2026 Feb 4;16(4):3628–36. doi: 10.1021/acscatal.5c08026 (PMC12930369; doi:10.1021/acscatal.5c08026)
Supplement: Supplementary file 1 [file cs5c08026_si_001.pdf]

Inactive but essential: The role of the inactive state of E49 in the mechanism of the alpha subunit of tryptophan synthase and its stand-alone blueprint *Zm*BX1

Cristina Duran,[a] and Sílvia Osuna*[a,b]

[a] Institut de Química Computacional i Catàlisi and Departament de Química, c/ Maria Aurèlia Capmany 69, 17003 Girona, Spain

E-mail: silvia.osuna@udg.edu

[b] ICREA, Pg. Lluís Companys 23, 08010 Barcelona, Spain

Supporting Information

Computational Methods:

Molecular modeling system preparation. The starting structures for the seven systems (*Zm*BX1, *Zm*TrpA, *Zm*TrpS, *Zm*TrpA^{L6BX1}, *Zm*TrpS^{L6BX1}, *Zm*TrpA$^{SPM4\text{-}L6BX1}$, *Zm*TrpS$^{SPM4\text{-}L6BX1}$) were generated with the multimer version of the AlphaFold2 (AF2)[1] neural network. The AF2 models simulated had a predicted LDDT-Cα score (pLDDT) higher than 92. To generate the version of the isolated TrpA systems, the alpha subunit from the TrpS model was extracted. For the homodimeric *Zm*BX1 enzyme, two X-ray structure are available (PDB codes: 1TJR and 1RD5) corresponding to two different dimerization modes. All *Zm*BX1 models generated were predicted to have the 1RD5 dimerization mode, thus it was used for running Molecular Dynamics (MD) simulations. The IGP substrate was placed in the TrpA subunits both in the isolated TrpA and the TrpA construct in complex through superposition to the G3P product in the Last Bacterial Common Ancestor TrpS X-ray structure (PDB code: 5EY5). For the heterocomplex MD simulations, the amino acrylate intermediate (A-A) was superimposed onto the same intermediate present in the *Salmonella typhimurium* TrpS X-ray structure (PDB code: 2J9X).

The water molecules added to each subunit were selected from the DBSCAN clusterization[2, 3] algorithm implemented in the scikit-learn Python library.[4] For TrpA, the water molecules of different TrpA subunits were used, ordered by PDB accession number and chain: 1TJR_A, 1TJR_B, 1RD5_A, 1RD5_B, 5EY5_A, 5EY5_C, 2DZS_A, 2DZS_B, 2DZU_A, 2DZU_B, 2E09_A, 2E09_B, 2DZW_A, 2DZW_B, 1GEQ_A, 1GEQ_B, 5E0K_A, 5E0K_C, 5E0K_E, 5E0K_G, 5E0K_I, 5E0K_K, 1WDW_A, 1WDW_C, 1WDW_E, 1WDW_G, 1WDW_I, 1WDW_K, 2DZT_A, 2DZT_B, 2DZV_A, 2DZV_B, 2DZP_A, 2DZP_B, 2DZX_A, 2DZX_B. In the MD simulations run on the IGP-bound state, the water molecules clashing with the substrate were removed manually. For the heterodimeric MD simulations, the water molecules of the following TrpB subunits were used, ordered by PDB accession number and chain: 5IXJ_A, 5IXJ_B, 5IXJ_C, 5IXJ_D, 5DW3_A, 5DW3_B, 5DW3_C, 5DW3_D, 5E0K_B, 5E0K_D, 5E0K_F, 5E0K_H, 5E0K_J, 5E0K_L, 5DW0_A, 5DW0_B, 5DW0_C, 5DW0_D, 1V8Z_A, 1V8Z_B, 1V8Z_C, 1V8Z_D, 5T6M_A, 5T6M_B, 5T6M_C, 5T6M_D, 1WDW_B, 1WDW_D, 1WDW_F, 1WDW_H, 1WDW_J, 1WDW_L, 5DVZ_A, 5DVZ_B, 5DVZ_C, 5DVZ_D, 6AMH_A, 6AMH_B, 6AMH_C, 6AMH_D, 6AMI_A, 6AMI_B, 6AMI_C, 6AMI_D, 6AMC_A, 6AMC_B, 6AMC_C, 6AMC_D, 5VM5_A, 5VM5_B, 5VM5_C, 5VM5_D, 6AM8_A, 6AM8_B, 6AM8_C, 6AM8_D, 6AM9_A, 6AM9_B, 6AM9_C, 6AM9_D, 6AM7_A, 6AM7_B, 6AM7_C, 6AM7_D, 6CUV_A, 6CUV_B, 6CUV_C, 6CUV_D, 6CUT_A, 6CUT_B, 6CUT_C, 6CUT_D, 6CUZ_A, 6CUZ_B, 6CUZ_C, 6CUZ_D, 5EY5_B, 5EY5_D. Additionally, one conserved sodium ion in the X-ray structure was added to the TrpB subunit located close to the active site.

The MD parameters for the substrate IGP and the A-A intermediate were generated with the antechamber and parmchk2 modules of AMBER20[5] using the 2nd generation of the general amber force-field (GAFF2).[5, 6] The IGP substrate and A-A intermediate were optimized at the B3LYP/6-31G(d) level of theory including Grimme's dispersion correction with Becke-Johnson Damping (D3-BJ) and the polarizable conductor model (PCM) (diethyl ether, ε = 4.2) as an estimation of the dielectric permittivity in the enzyme active site.[7] The partial charges (RESP model)[8] were set to fit the electrostatic potential

generated at the HF/6-31G(d) level of theory. The charges were calculated according to the Merz-Singh-Kollman[9] scheme using the Gaussian16 software package.[10] The protonation states were predicted using PROPKA.[11] For *Zm*BX1, *Zm*TrpA, *Zm*TrpA^{L6BX1} and *Zm*TrpA$^{SPM4\text{-}L6BX1}$ the protonation state of the catalytic residue E49/50 was neutral (i.e., GLH49 and GLH50), as is described in the TrpA mechanism. For the heterocomplex simulations, the protonation state of the TrpB catalytic residue K84 was neutral (i.e., LYN84), as is described in the mechanism.[12] The enzyme structures were solvated in a pre-equilibrated truncated octahedral box of 10 Å edge distance using the OPC water model and neutralized by the addition of explicit counterions (i.e., Na^+) using the AMBER20 leap module. All MD simulations were performed using a modification of the amber99 force field (ff19SB).[13]

MD simulation details. MD equilibration phase was done following the protocol described by Roe and Brooks with small differences fine-tuned to our systems.[14] The bonds involving hydrogen are constrained by the SHAKE algorithm during the non-minimization steps. Long-range electrostatic effects were modeled using the particle mesh-Ewald method.[15] For Lennard–Jones and electrostatic interactions, a 10 Å cut-off was applied. The MD protocol starts with the minimization phase of 1500 steps of the steepest descent method followed by 3500 steps of the conjugate gradient method with a positional restrain (*i.e.*, a force constant of 5.0 kcal·mol^{-1}·Å^{-2}) to the protein heavy atoms. In the following heating phase a temperature increment from 25 K to 300K during 20 ps of MD simulation time, a Langevin thermostat with a collision frequency of 5 ps^{-1}, and a positional restrain (i.e., a force constant of 5.0 kcal·mol^{-1}·Å^{-2}) to the protein heavy atoms; are performed. A minimization and heating of all atoms in the system is the following step. This starts with two minimization stages of 1000 steps of the steepest descent method followed by 1500 steps of the conjugate gradient method each with a positional restrain (i.e., force constant of 2.0 kcal·mol^{-1}·Å^{-2} in the first minimization and 0.1 kcal·mol^{-1}·Å^{-2} in the second) to the protein heavy atoms. Following, a third minimization phase of 1500 steps of the steepest descent method followed by 3500 steps of the conjugate gradient method without any positional restraint is performed. The system is then heated in accordance with the previously established procedure. Finally, a five-round equilibration phase at the NPT ensemble with a constant pressure of 1 atm is performed. The first four rounds were done with the Berendsen barostat, whereas the fifth one was done with a Monte-Carlo barostat. For all equilibration rounds, Langevin thermostat with a collision frequency of 1 ps^{-1} was used. A positional restraint to the protein-heavy atoms with a force constant of 1.0 and 0.5 kcal·mol^{-1}·Å^{-2} was applied to the first and second equilibration rounds, respectively. In the third round of 10 ps equilibration, a positional restraint to the backbone-heavy atoms with a force constant of 0.5 kcal·mol^{-1}·Å^{-2} was used. The fourth and fifth equilibration of 10 ps and 1 ns, respectively, were performed without any restraint. The production runs were performed at the NVT ensemble with the Langevin thermostat with a collision frequency of 1 ps^{-1} during 500 ns for all TrpA and *Zm*BX1 systems, and 400 ns for TrpS complexes. A total of 10 replicas of equilibration and production runs were performed reaching a total simulation time of 5 µs/system (10 replicas x 500 ns) for *Zm*BX1, *Zm*TrpA, *Zm*TrpA^{L6BX1}, *Zm*TrpA^{SPM4}, *Zm*TrpA^{SPM6} and *Zm*TrpA$^{SPM4\text{-}L6BX1}$ systems. For the heterocomplexes (*i.e.*, *Zm*TrpS, *Zm*TrpS^{L6BX1} and *Zm*TrpS$^{SPM4\text{-}L6BX1)}$) 6 replicas of equilibration and production runs were performed reaching a total simulation time of 2.4 µs for each system (6 replicas

x 400 ns). The MD trajectories were analyzed using the Python packages MDTraj,[16] pytraj[17] which is part of the cpptraj package,[18] MDAnalysis,[19] and PyEMMA.[20]

Free Energy Landscape (FEL) reconstruction. Molecular dynamics (MD) simulations allow the sampling of the population distribution of biomolecules by integrating Newton's laws of motion. This process enables the recovery of thermodynamic properties such as the free energy. However, due to the vast number of atoms involved in the MD simulations, this probability distribution of molecular states is represented in an extremely high-dimensional space. This is usually solved by focusing on a selected set of degrees of freedom (DOF) relevant to the process of interest. In our case we used the dihedral χ_1 of the catalytic E49 (Figure 2), the catalytic distance between the carboxylate carbon of E49 and the 3' hydroxyl of IGP (Figures 2 and 5), and the distance between Tyr58 and Asp125 for the closed-to-open transition of L2 (Figure 5). High dimensional data obtained from MD simulations can be projected onto these DOFs for obtaining the probability distributions and reconstructing the free energy (eq. 1).

$$G \sim -k_B T \log (P) \qquad \text{(eq. 1)}$$

where the free energy (G) is defined as the negative logarithm of the population distribution (P) in $k_B T$ units (e.g. kcal/mol·K). A maximum in the distribution corresponds to a minimum in the free energy surface.

Quantum Mechanical (QM) calculations. The cluster models were obtained from the X-ray structure of *Zm*BX1 (PDB code: 1TJR), composed by the following residues: E49, I59, D60, L99, Y101, V123, P124, Y170, V172, T178, F207, G208, I228, G229. For the Asp60Asn mutation, the same cluster model was used, incorporating the Asn60 in the same conformation of the X-ray structure of this single mutant variant (PDB code: 1A5B) All the optimization and high-level energy calculations were performed with Gaussian16[10]. The systems were described with B3LYP functional with the GD3 dispersion correction[21] and adding solvation corrections through the Solvation Model based on Density (SMD).[22] 6-31G* was used as basis set. All energies were calculated by performing single-point calculations on the optimized geometries using the functional ωB97XD[23] with the 6-311+G(2d,2p) basis set. All the computational data obtained from the single-point calculations have been uploaded onto the IOCHEM-BD platform (https://doi.org/10.19061/iochem-bd-4-86).

Quantum Mechanics/Molecular Mechanics (QM/MM) calculations. The initial enzyme-substrate complex was constructed from the available X-ray crystal structure *Zm*BX1 (PDB code: 1TJR). All QM/MM calculations were performed using the ONIOM method[24] as implemented in Gaussian16[10]. Input preparation, including definition of the QM region and specification of the link atoms, was carried out using MolUP VMD extension,[25] which provides an automated and reproducible framework for constructing models and defining QM/MM boundaries. Link atoms along the QM/MM boundary were defined automatically in MolUP using the standard hydrogen link-atom scheme.

The QM region comprised the catalytically relevant residues E49, D60, and Y170, together with the IGP molecule (62 atoms and 3 H-link atoms). The remainder of the enzyme and solvent molecules were treated at the MM level using the AMBER ff14SB[26] force field. All residues and water molecules around 10 Å form the QM region are

constituting the active region, being free to move during optimization steps. A two-step optimization protocol was employed using QuadMacro algorithm. First, mechanical embedding (ME) optimizations were performed and subsequently, electrostatic embedding (EE) optimizations were conducted while maintain water molecules frozen, accounting for the polarization of the QM region by the surrounding enzyme environment.[27] The QM region was treated at the B3LYP/6-31G* level during geometry optimizations. All energies were calculated by performing single-point calculations on the EE optimized geometries using the functional ωB97XD[23] with the 6-311+G(2d,2p) basis set. All transition states were characterized by harmonic frequency analysis and confirmed with a single imaginary frequency. Reported relative energies correspond to single-point ONIOM(ωB97X-D/6-311+G(2d,2p):AMBER) energies computed on the optimized ONIOM(B3LYP/6-31G*:AMBER) geometries.

Water analysis. Water positions and conservation are analyzed using CuPy to compute top-K nearest-neighbor anchors, the Kabsch algorithm for rigid-body alignment, and cuML DBSCAN to detect hydration clusters.[2, 28] Conservation is defined as the fraction of frames occupied by a cluster, and centroids are calculated from aligned water positions. MDAnalysis is used to analyze molecular dynamics trajectories.[19]

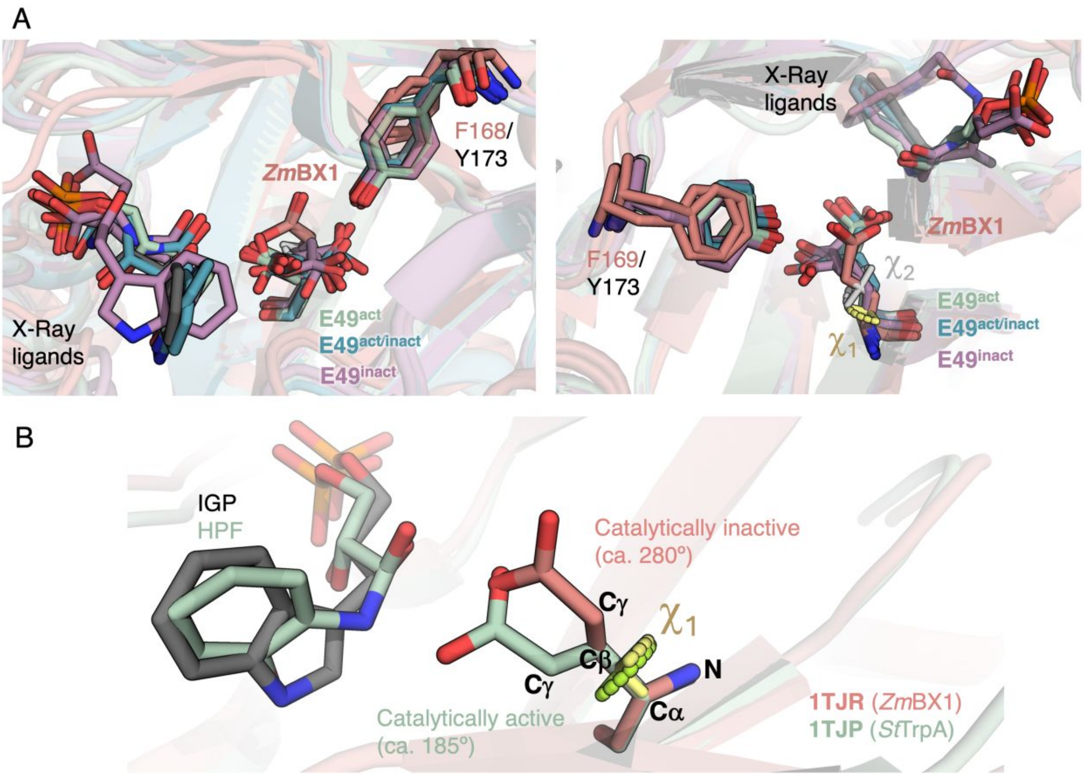

Figure S1. Overlay of *Zm*BX1 and TrpA X-ray structures. A. *Zm*BX1 adopts a different inactive state of E49 that differs from the reported TrpA conformation. The main difference lies in the χ_1 dihedral of E49 (280º versus ca. 210º in *Zm*BX1, and TrpAs, respectively). For the X-ray structures of TrpA, the active and inactive states differ in the χ_2 dihedral of E49 (73º for the inactive state and 196º for the active), mostly due to the interaction with Tyr173. *Zm*BX1 is shown in pink, TrpAs with E49 in active state are shown in green, TrpAs with E49 in inactive state are shown in purple, and TrpAs displaying both E49 states are shown in blue. **B.** *Zm*BX1 and *St*TrpA (complexed with 1-[(2-hydroxylphenyl)amino]3-glycerolphosphate, HPF) show two different conformations of E49 χ_1 dihedral angle, which is described by N-Cα-Cβ-Cγ atoms.

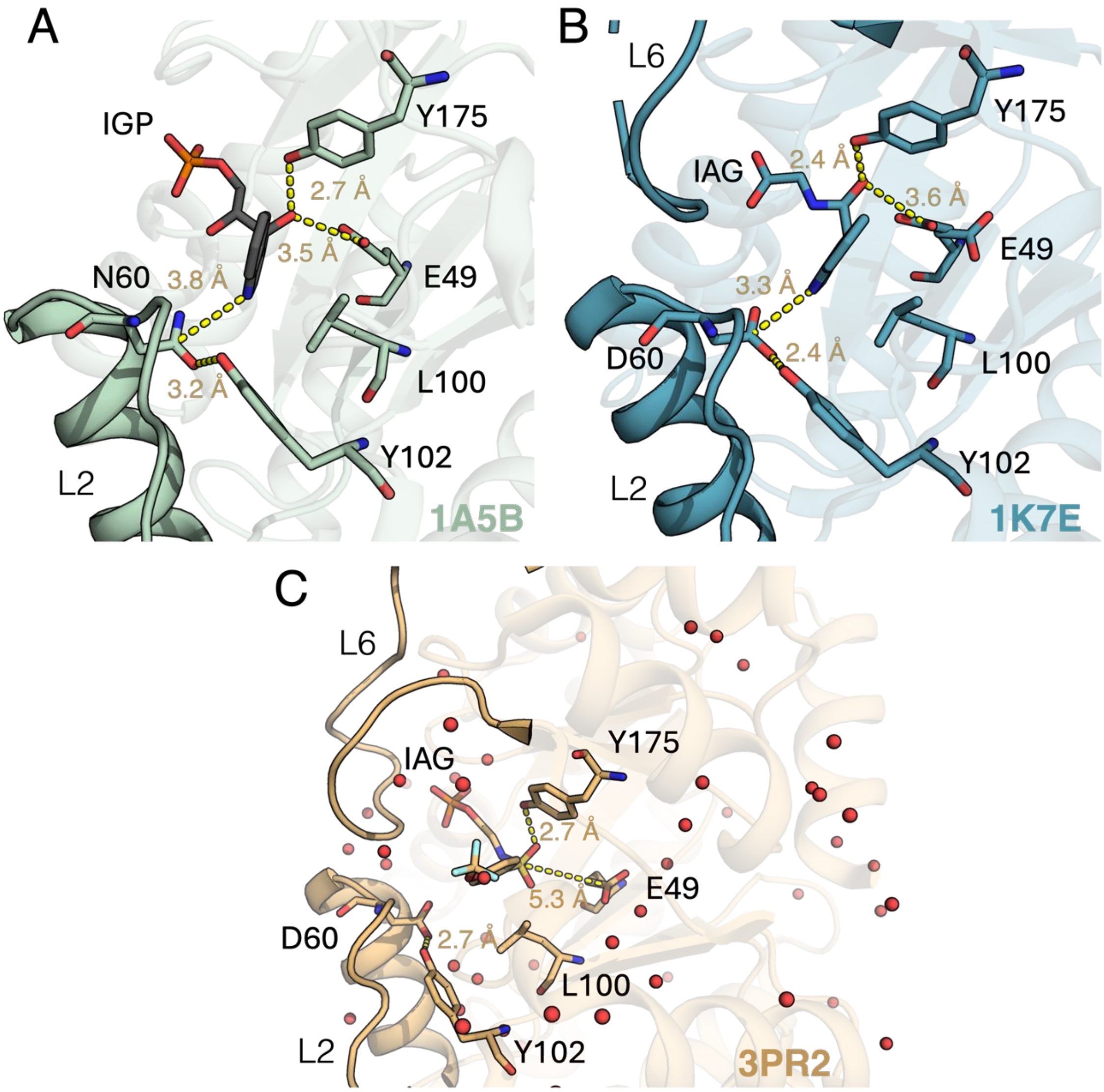

Figure S2. Representation of several TrpA X-ray structures. A. The X-ray structure of the *Salmonella typhimurium* TrpA (*St*TrpA) inactive Asp60Asn variant shows that the active orientation of E49 is the one adopted in the presence of the true IGP substrate (PDB: 1A5B). **B.** X-ray structure of *St*TrpA with indole-3-acetylglycine bound (IAG, PDB: 1K7E), E49 establishes a hydrogen bond with the acetyl oxygen of IAG, thus indicating that E49 is protonated under physiological conditions. **C.** X-ray structure used for conducting QM/MM studies[29] that does not have any crystallographic water between the catalytic E49 and IGP analogue (PDB: 3PR2).

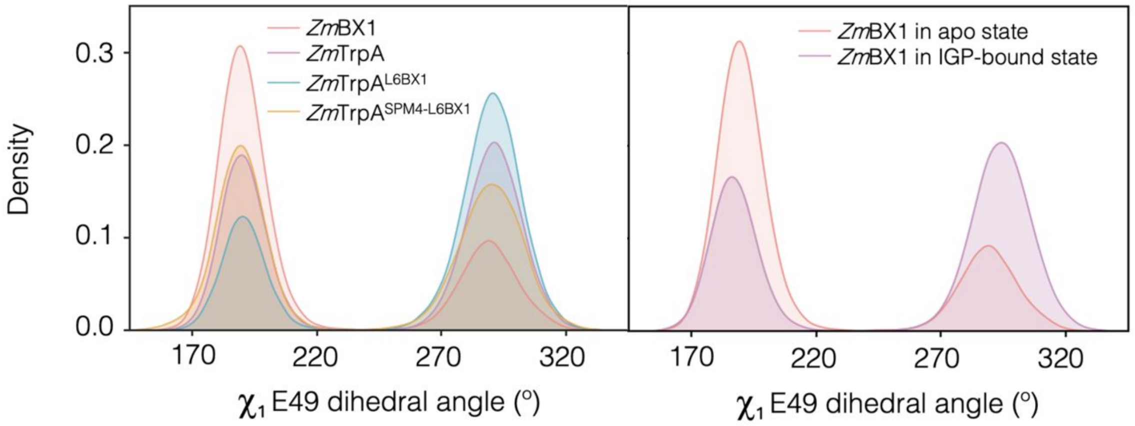

Figure S3. Histogram of the χ_1 dihedral of the catalytic E49. Left. Histogram in the apo state for all systems. *Zm*BX1 is shown in pink, isolated *Zm*TrpA in purple, isolated *Zm*TrpA^{L6BX1} in blue, and *Zm*TrpA$^{SPM4\text{-}L6BX1}$ in orange. Active states of E49 present χ_1 of ca. 185º, whereas inactive states values of 290º. The histogram shows a progressive destabilization of the active state along the series: *Zm*BX1< *Zm*TrpA$^{SPM4\text{-}L6BX1}$ < *Zm*TrpA < *Zm*TrpA^{L6BX1}. **Right**. The histogram of *Zm*BX1 in the apo and IGP-bound states is represented.

A. *Zm*BX1 in apo state

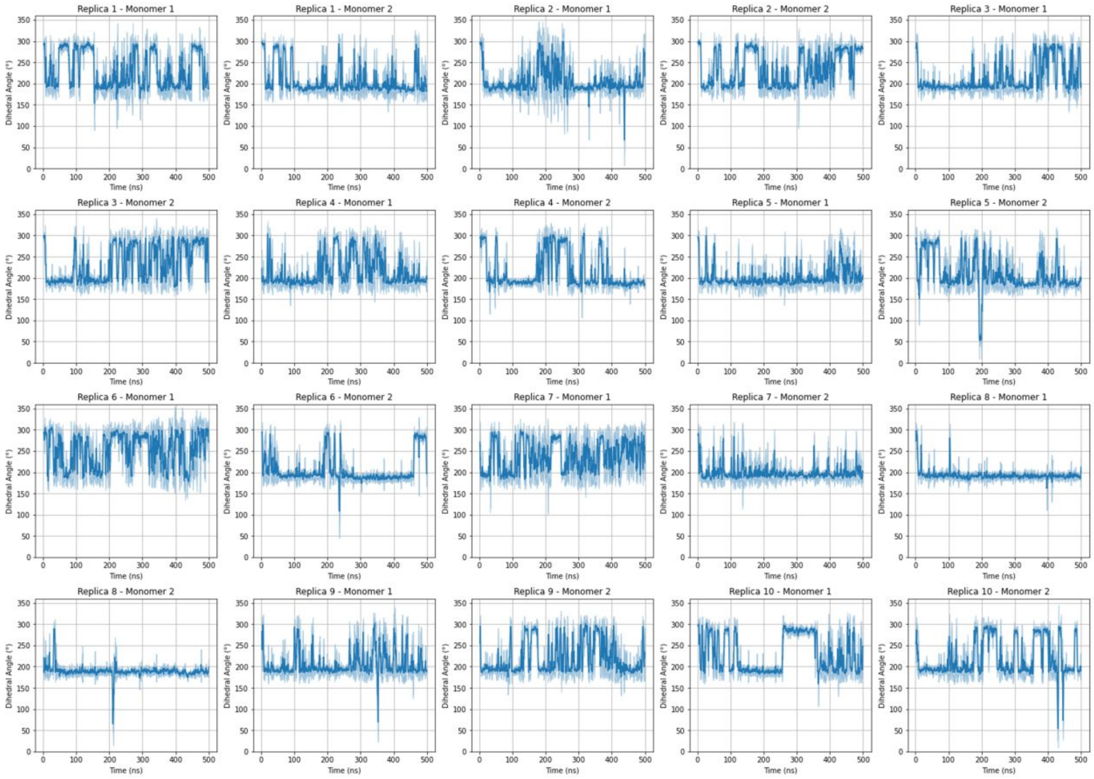

B. *Zm*BX1 in IGP-bound state

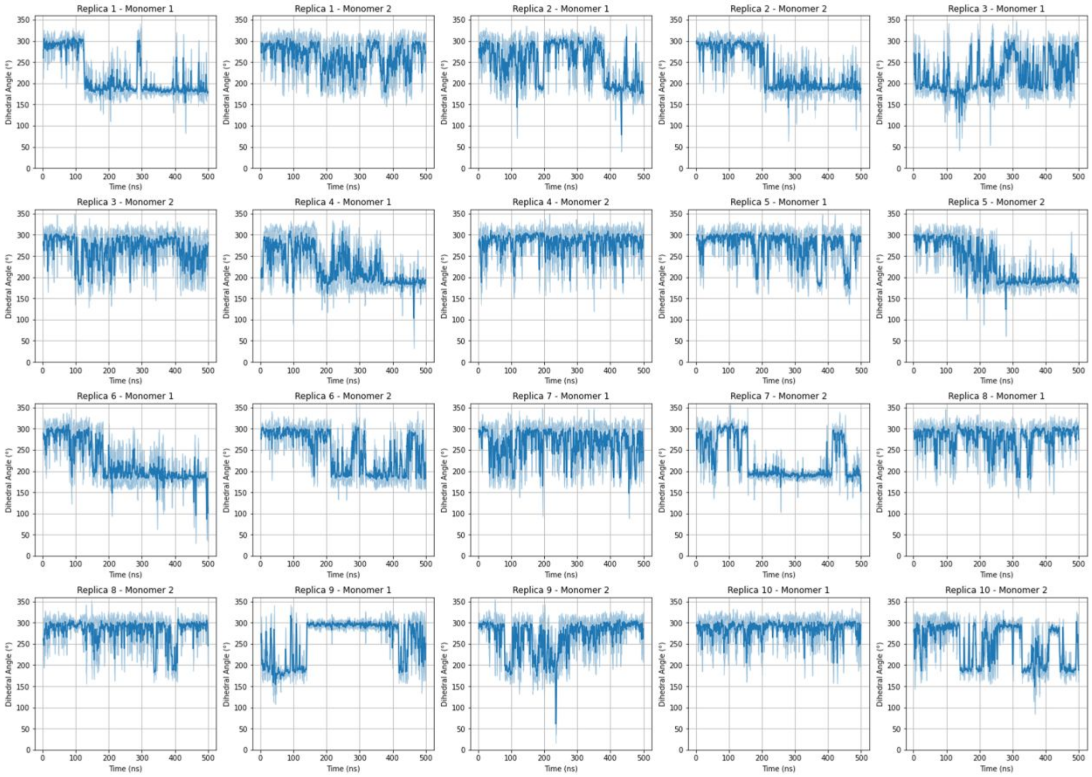

Figure S4. Plot of the E49 dihedral along the 500 ns MD simulation time for each replica and monomer of *Zm*BX1. A. apo state. **B.** IGP-bound state. The dihedral is in degrees.

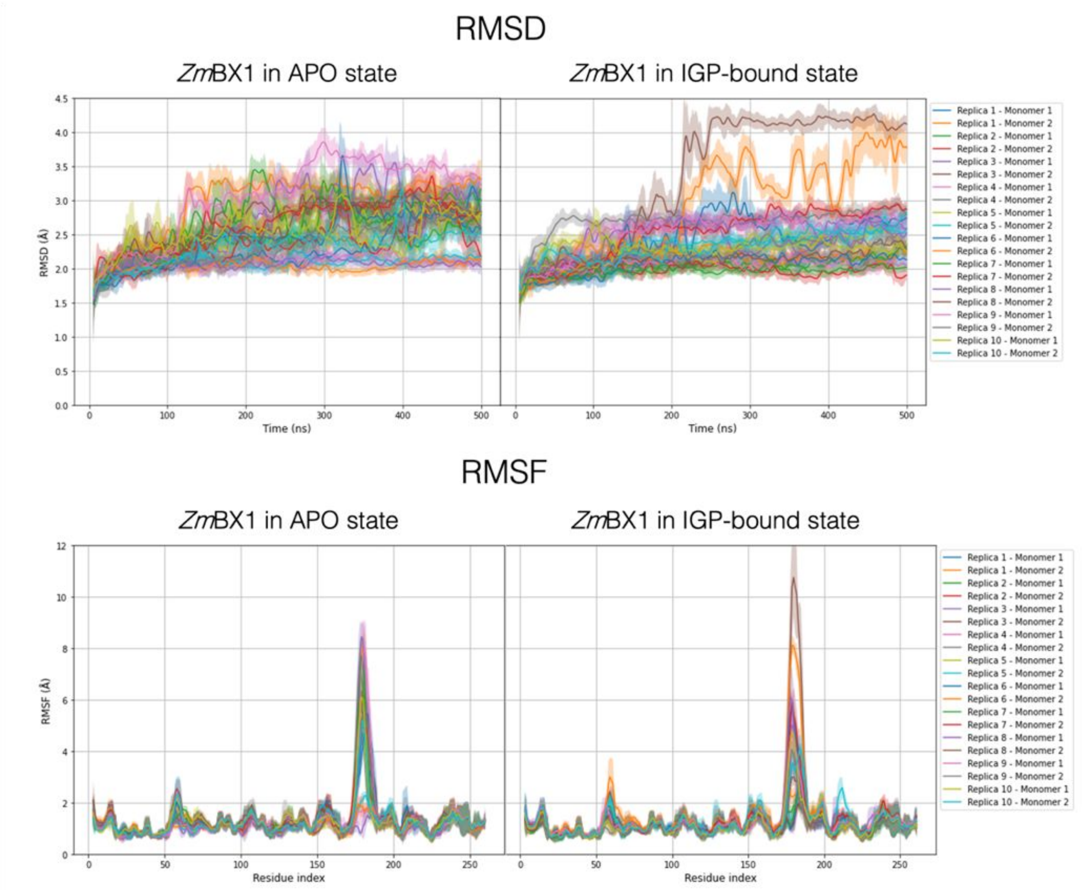

Figure S5. Root Mean Square Deviation (RMSD) and Fluctuation (RMSF) for *Zm*BX1 in the apo and IGP-bound states. Each replica is represented using a different color.

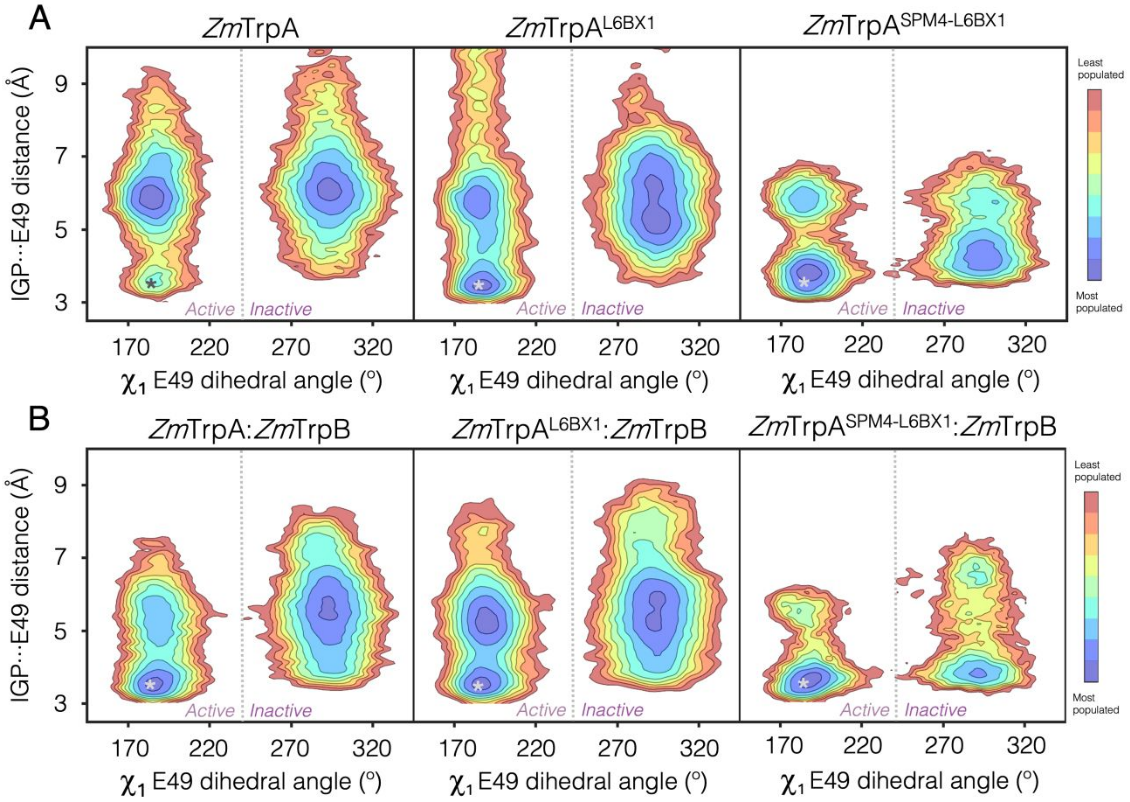

Figure S6. Conformational landscapes of several IGP-bound *Zm*TrpAs in the absence (A) and presence of TrpB (B). The reconstructed conformational landscapes are based on the dihedral χ_1 of the catalytic E49 (x-axis) and the catalytic distance between the carboxylate carbon of E49 and the 3' hydroxyl of IGP (y-axis). Active states of E49 present χ_1 of ca. 185º, whereas inactive states values of 290º. For comparison the crystallographic values obtained for the IGP-bound structure of the Asp60Asn mutant are represented with asterisks. Most stable conformations are colored in blue, whereas least stable ones in red. Short IGP-E49 distances (< 4 Å) are stabilized in those systems presenting a high catalytic activity: all *Zm*TrpA in complex with *Zm*TrpB (B panel), and the rationally designed *Zm*TrpA$^{SPM6-L6BX1}$ (A right panel), substantially longer distances (ca. 6 Å) are favored for isolated *Zm*TrpA (shown in A left panel) and *Zm*TrpA^{L6BX1} (A middle panel) that have poor stand-alone catalytic activities.[30]

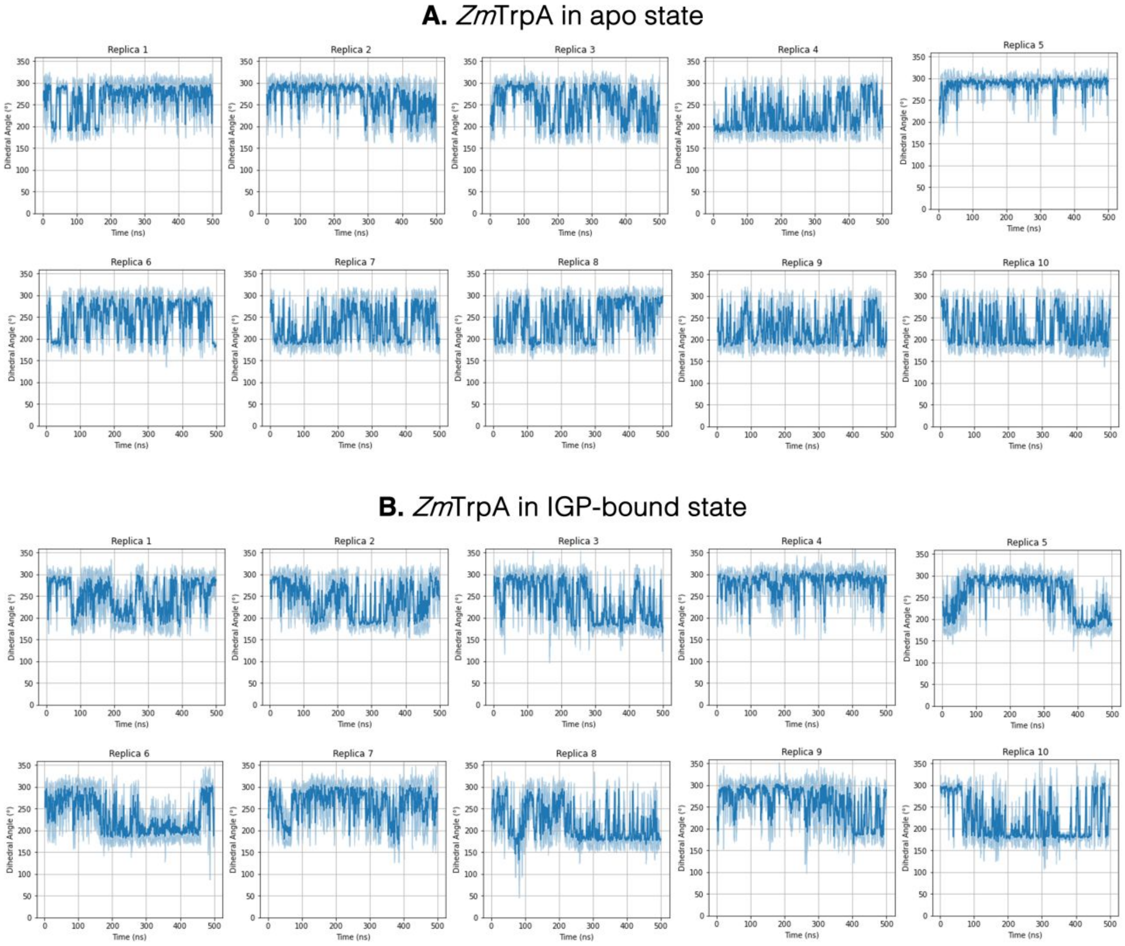

Figure S7. Plot of the E49 dihedral along the 500 ns MD simulation time for each replica and monomer of *Zm*TrpA. A. apo state. **B.** IGP-bound state. The dihedral is in degrees.

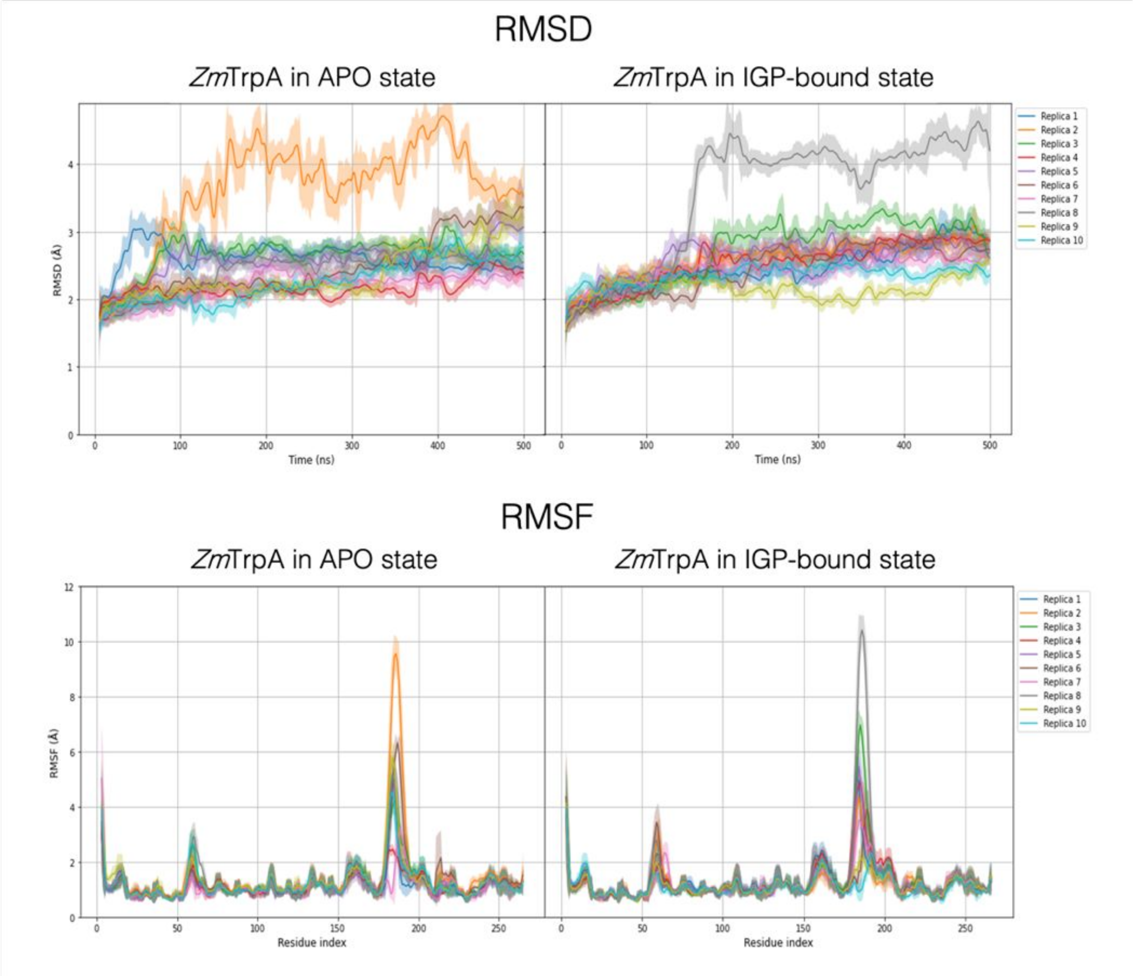

Figure S8. Root Mean Square Deviation (RMSD) and Fluctuation (RMSF) for isolated *Zm*TrpA in the apo and IGP-bound states. Each replica is represented using a different color.

Table S1. Summary of the reported kinetic constants for *Zm*BX1, *Zm*TrpA and variants in the absence (stand-alone) and presence of TrpB (in complex).

Protein	k_{cat} [s^{-1}]	K_M [M]	k_{cat}/K_M [M^{-1}s^{-1}]	Fold increase
*Zm*BX1	5.2 ± 0.13	11 ± 1.3	474044 ± 56093	-
*Zm*TrpA	0.005 ± 0.001	1530 ± 327	3.3 ± 0.8	-
*Zm*TrpA + *Zm*TrpB	2.9 ± 0.1	195 ± 17.9	15006 ± 1430	4515[b]
*Zm*TrpA^{L6BX1}	1.2 ± 0.09	3351 ± 340	355 ± 45.4	108[a]
*Zm*TrpA^{L6BX1} + *Zm*TrpB	0.35 ± 0.013	111 ± 14.1	3106 ± 410	8.9[b]
*Zm*TrpA$^{SPM4-L6BX1}$	0.59 ± 0.03	1110 ± 105	533 ± 56	163[a]
*Zm*TrpA$^{SPM4-L6BX1}$ + *Zm*TrpB	0.29 ± 0.01	70 ± 12	4080 ± 698	6.5[b]

[a]Fold TrpA activity increase in terms of k_{cat}/K_M of each variant alone compared to ZmTrpA.
[b]Fold activation in terms of k_{cat}/K_M of each variant by ZmTrpB

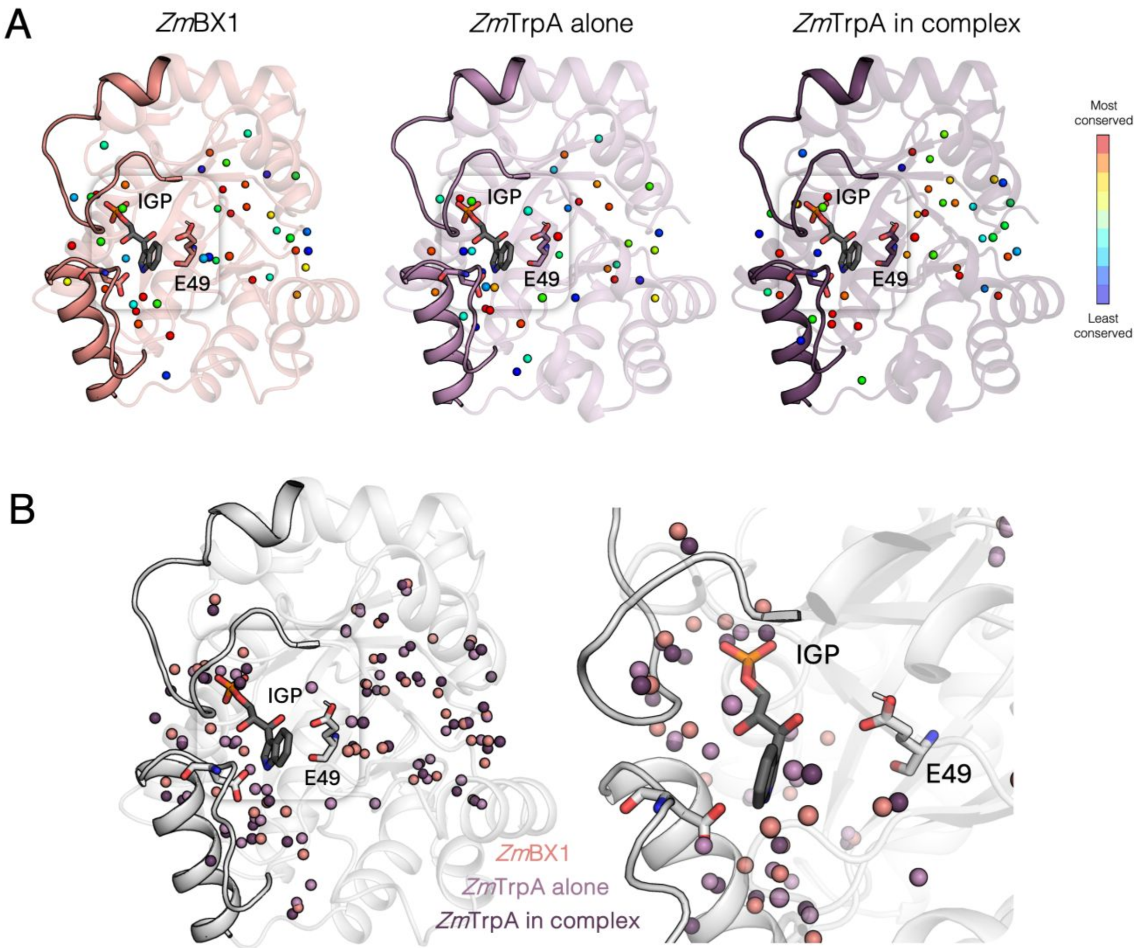

Figure S9. Water clusterization analysis of *Zm*BX1, *Zm*TrpA alone and in complex with ZmTrpB. A. Water cluster for (from left to right) *Zm*BX1, *Zm*TrpA alone and in complex with ZmTrpB. Most conserved water molecules (*i.e.*, a higher percentage of frames have the specific water in the corresponding position) are colored in red whereas least conserved ones, in blue. **B.** Overlay of the water clusters of all systems: water molecules observed in *Zm*BX1 are shown in coral, *Zm*TrpA alone in light purple, and in complex with ZmTrpB, in purple. The active site zoom (right panel B) shows that any water molecule is positioned between E49 and the IGP substrate in any of the analyzed systems.

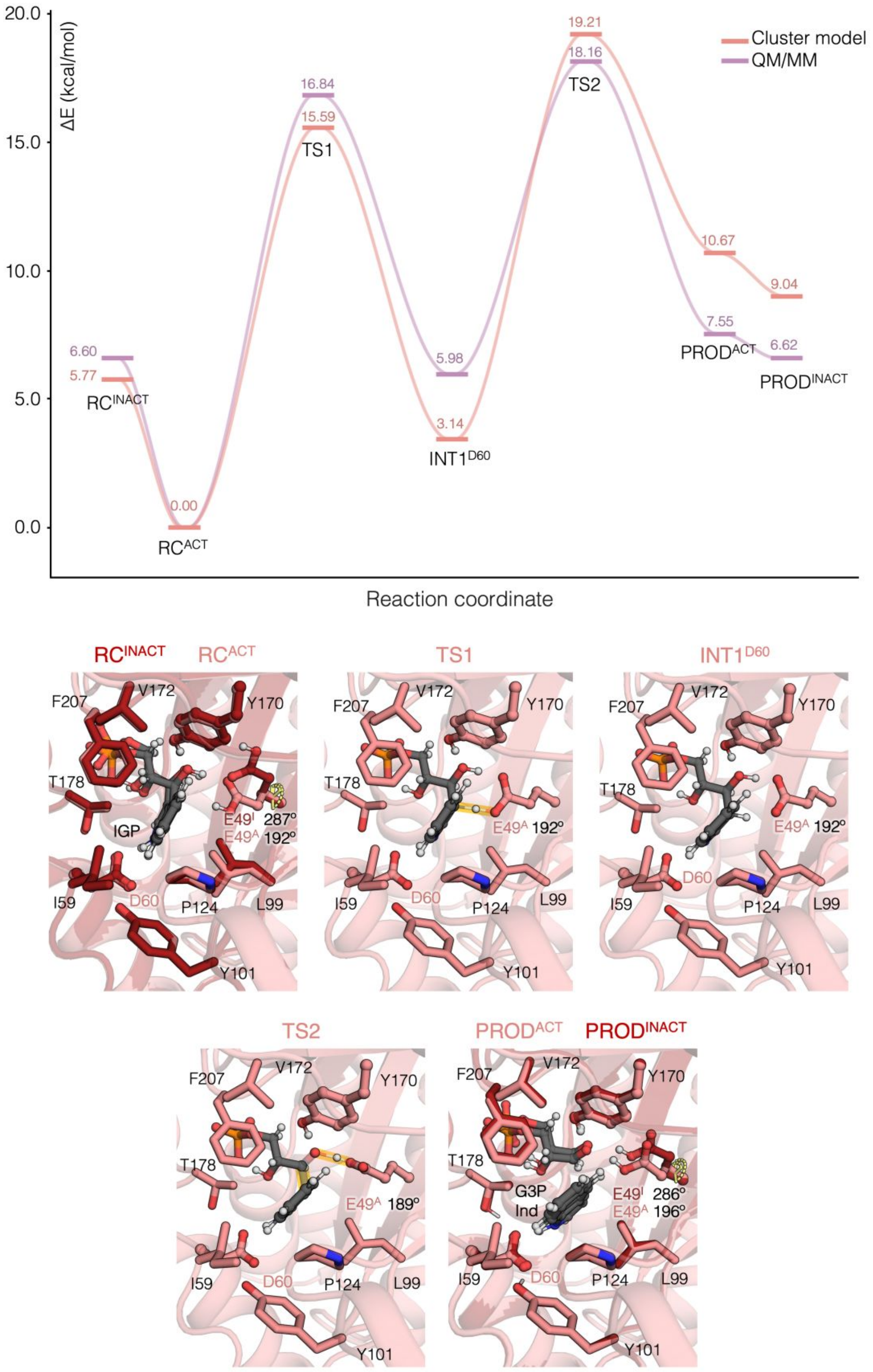

Figure S10. QM/MM and cluster model reaction mechanism of E49 and D60 catalyzed retro-aldol cleavage in TrpA/*Zm*BX1. The computed reaction profile for the QM/MM calculations is shown in pink, whereas the mechanism obtained performing QM/MM calculations is represented in purple. All the optimized structures obtained with QM/MM along the reaction coordinate are shown at the bottom. The residues defining the QM region (E49, D60, Y170 and IGP) are highlighted in spheres. IGP is shown as grey spheres. The residues included in the cluster model calculations are show in sticks as a comparison. See Tables S1 and S2 to compare the key distances of the transition states geometries (TS1 and TS2) obtained in the cluster model and QM/MM calculations.

QM/MM calculations confirm the conclusions obtained from the cluster model calculations.

Table S2. Comparison of the key distances and frequency describing the first transition state (TS1) of E49 and D60 catalyzed retro-aldol cleavage in TrpA/*Zm*BX1 using cluster model and QM/MM calculations. All distances are shown in Å.

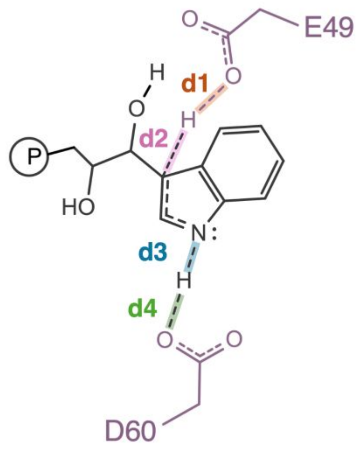

TS1: indole C3 protonation

	Cluster model distance (Å)	QM/MM distance (Å)
**d1** (E49) O ··· H (O-E49)	1.253	1.239
**d2** (IGP-O) H ··· O (IGP)	1.411	1.427
**d3** (IGP) N1 ··· H (N1-IGP)	1.041	1.058
**d4** (IGP-N1) H ··· O (D60)	1.773	1.675

	Cluster model	QM/MM
Frequency (cm^{-1})	-1555.1	-1196.2

Table S3. Comparison of the key distances and frequencies describing the second transition state (TS2) of E49 and D60 catalyzed retro-aldol cleavage in TrpA/*Zm*BX1 using cluster model and QM/MM calculations. All distances are shown in Å.

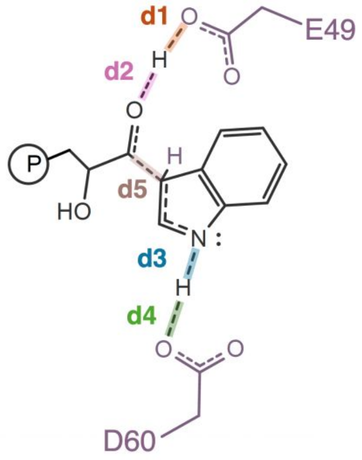

TS2: C-C bond breaking (retro-aldol cleavage)

	Cluster model distance (Å)	QM/MM distance (Å)
**d1** (E49) O ··· H (O-IGP)	1.032	1.030
**d2** (IGP-O) H ··· O (IGP)	1.545	1.494
**d3** (IGP) N1 ··· H (N1-IGP)	1.051	1.087
**d4** (IGP-N1) H ··· O (D60)	1.659	1.545
**d5** (IGP) C4 ··· C3 (IGP)	1.978	1.699

	Cluster model	QM/MM
Frequency (cm^{-1})	-384.3	-374.3

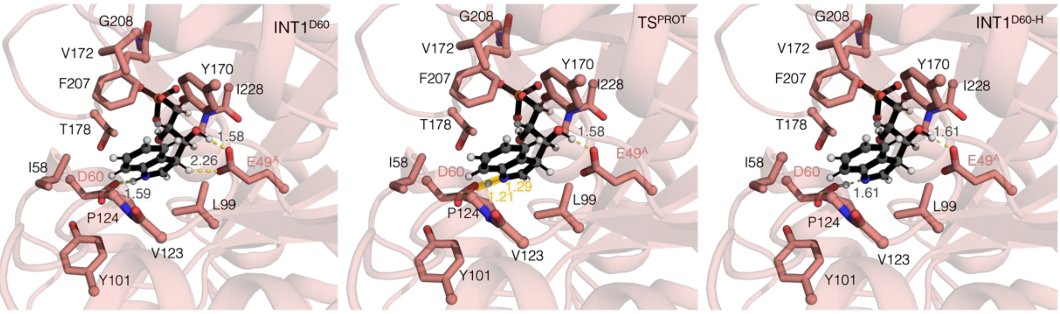

Figure S11. DFT optimized structures of INT1^{D60}, TS^{PROT}, and INT1$^{D60\text{-}H}$. The most relevant distances are shown in Å. IGP is shown as grey spheres and black sticks. The atoms kept frozen during the optimization are marked with a pink sphere. The transition state for N1 (de)protonation is 1 kcal/mol, both INT1s present a similar stability of ca. 0.8 kcal/mol higher than RC^{ACT}.

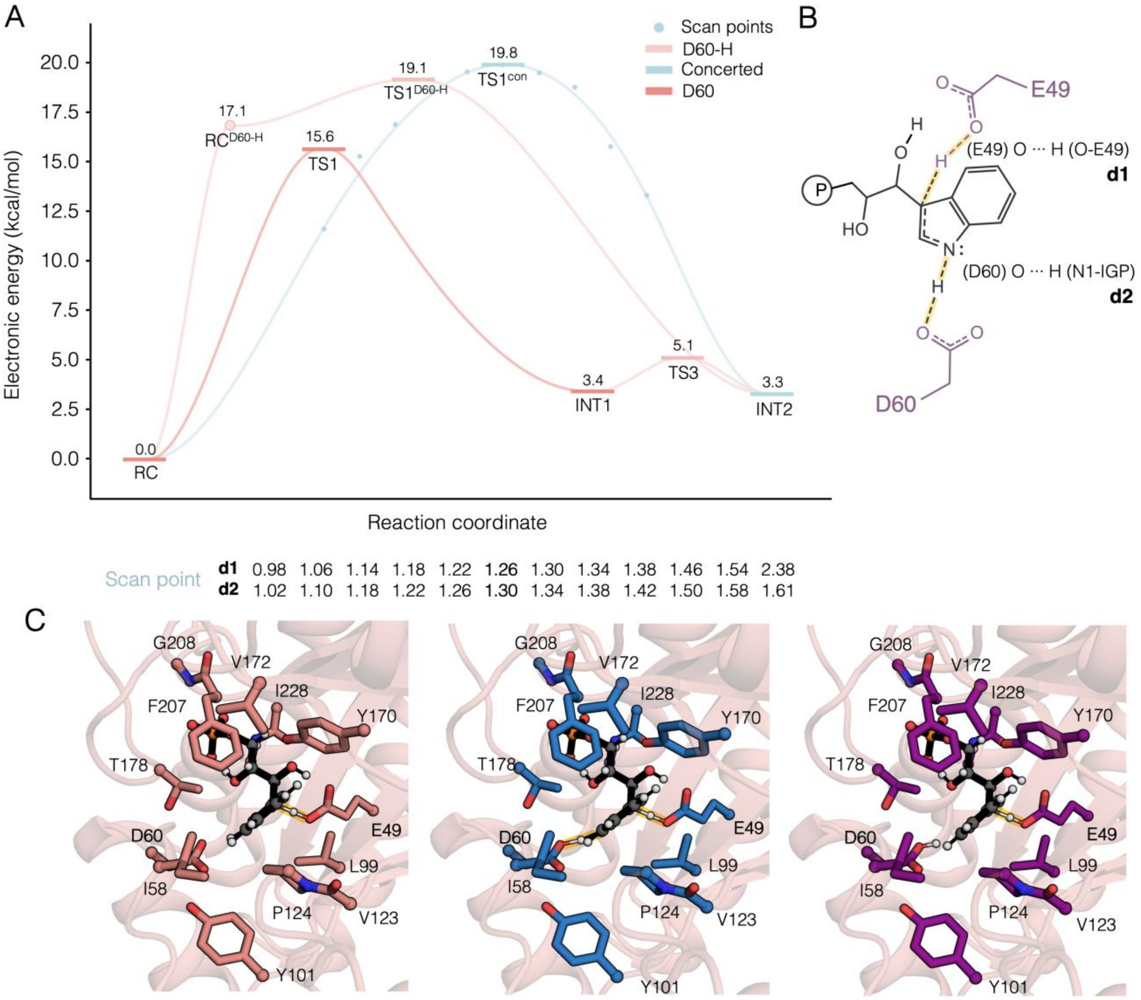

Figure S12. A. Representation of the reaction coordinate for the concerted TS^{CON} and stepwise TSs considering both D60 protonated or deprotonated (TS1, TS1$^{D60\text{-}H}$). For the concerted TS^{CON} a scan based on the two relevant distances shown in (**B**) of the potential energy surface was performed. All attempts to locate this TS^{CON} starting from the maximum along the RC yielded the lowest in energy stepwise TS1. Our estimated upper-level activation energy for TS^{CON} and also the located TS1$^{D60\text{-}H}$ are ca. 9 kcal/mol higher in energy than for TS1. **C.** DFT optimized structures of TS1 (pink), estimated TS^{CON} based on the scan (blue), and optimized TS1$^{D60\text{-}H}$ (purple).

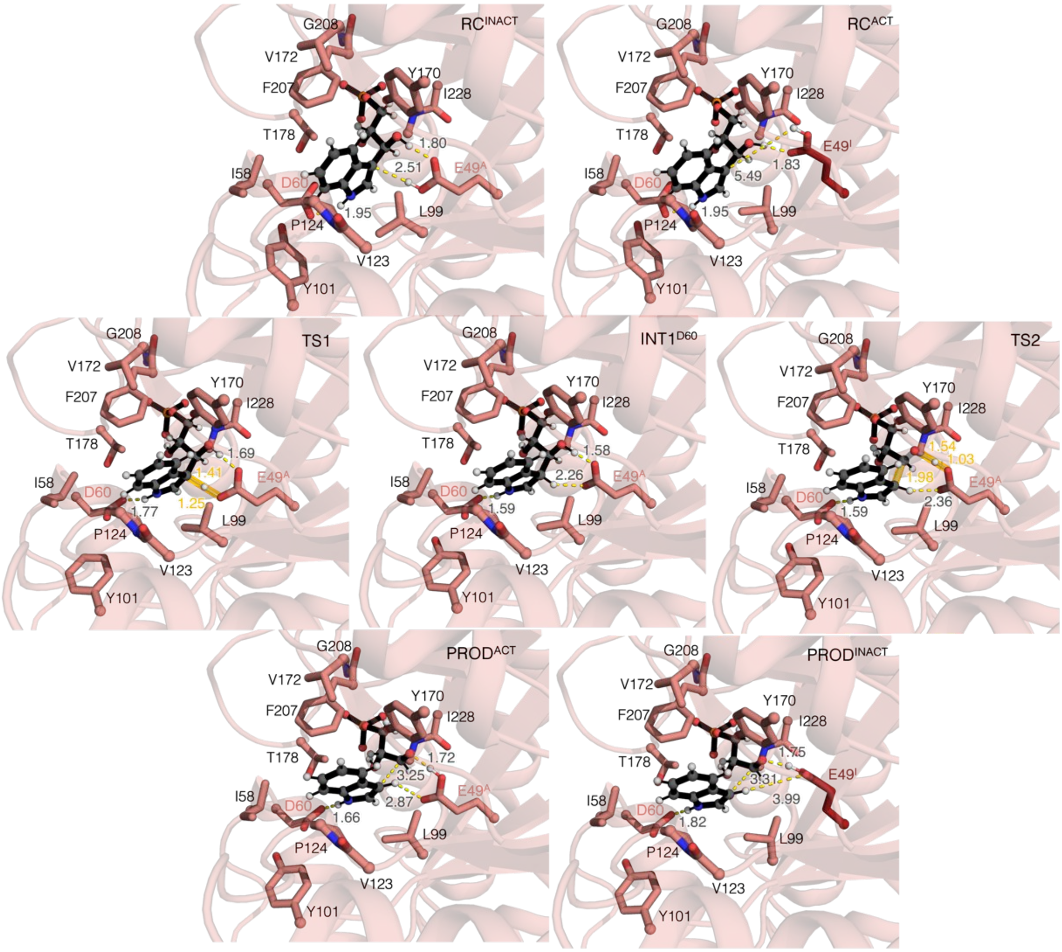

Figure S13. DFT optimized structures of all RC, intermediates and products for wild-type TrpA/*Zm*BX1. The most relevant distances are shown in Å. IGP is shown as grey spheres and black sticks. The atoms kept frozen during the optimization are marked with a pink sphere.

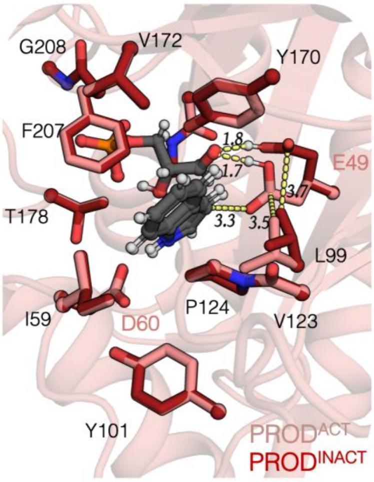

Figure S14. Overlay of the DFT optimized structures of E·P complexes presenting E49 in the active and inactive state for *Zm*BX1.

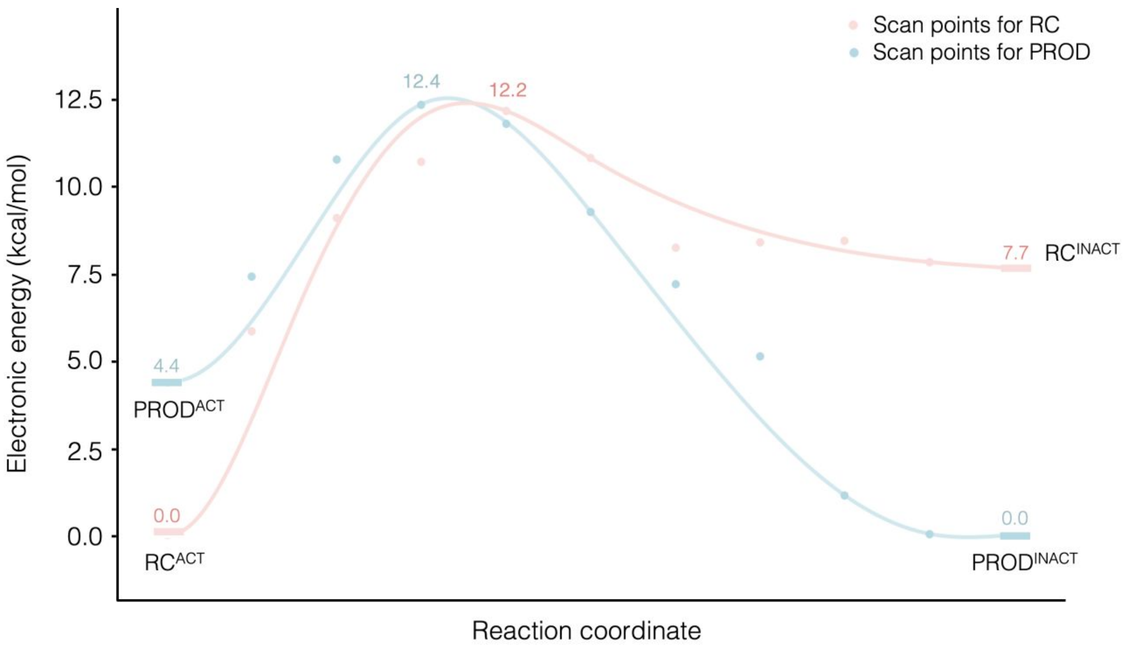

Scan point: χ_1 E49 dihedral angle (°)												
RC^{ACT}	185	196	206	216	**226**	236	246	256	266	276	287	RC^{INACT}
PROD^{ACT}	183	195	206	**217**	228	239	250	261	272	283	295	PROD^{INACT}

Figure S15. Scan of the dihedral χ_1 of the catalytic E49 for the inactive-to-active transition of E49 at the E·S and E·P complexes. Our estimated upper-level activation energy for the transition is ca. 12.5 kcal/mol.

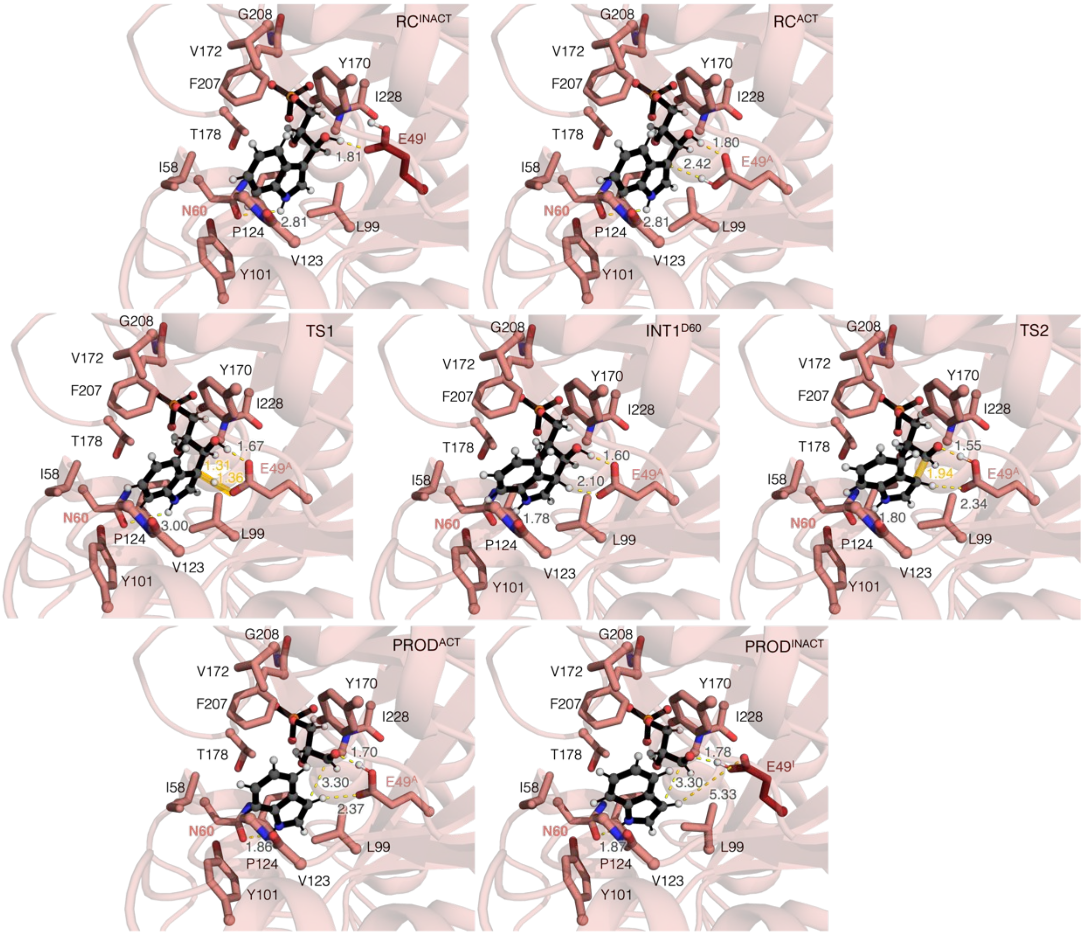

Figure S16. DFT optimized structures of all RC, intermediates and products for D60N variant. The most relevant distances are shown in Å. IGP is shown as grey spheres and black sticks. The atoms kept frozen during the optimization are marked with a pink sphere.

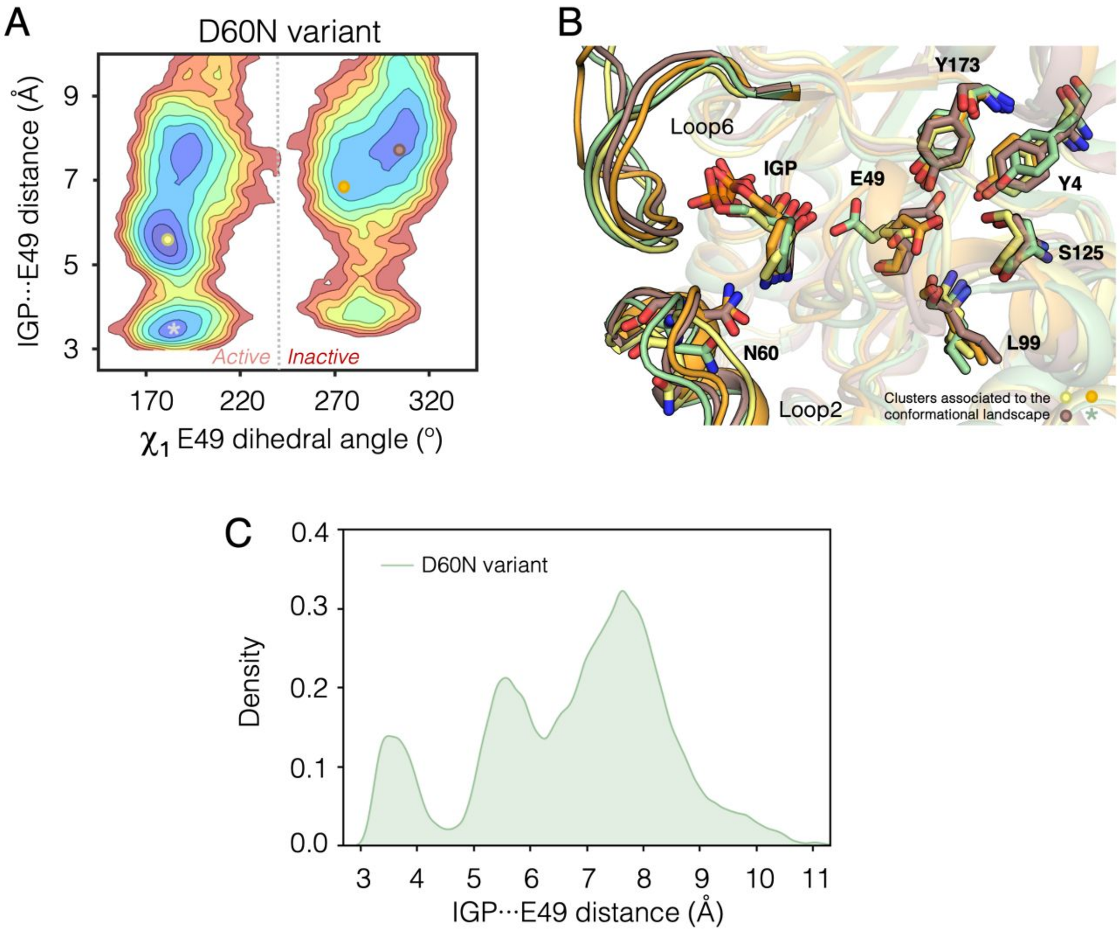

Figure S17. MD simulations of the D60N variant at the IGP-bound state. A. Reconstructed conformational landscape based on the dihedral χ_1 of the catalytic E49 (x-axis) and the catalytic distance between the carboxylate carbon of E49 and the 3' hydroxyl of IGP (y-axis). Active states of E49 present χ_1 of ca. 185º, whereas inactive states values of 290º. For comparison the crystallographic value is represented with asterisks. Most stable conformations are colored in blue, whereas least stable ones in red. Different structures are extracted from different regions of the landscape, highlighted in colored circles. **B.** Overlay of the corresponding structures extracted from the conformational landscape. Each structure color is associated to the circle color in panel A. **C.** Histogram of the χ_1 dihedral of the catalytic E49.

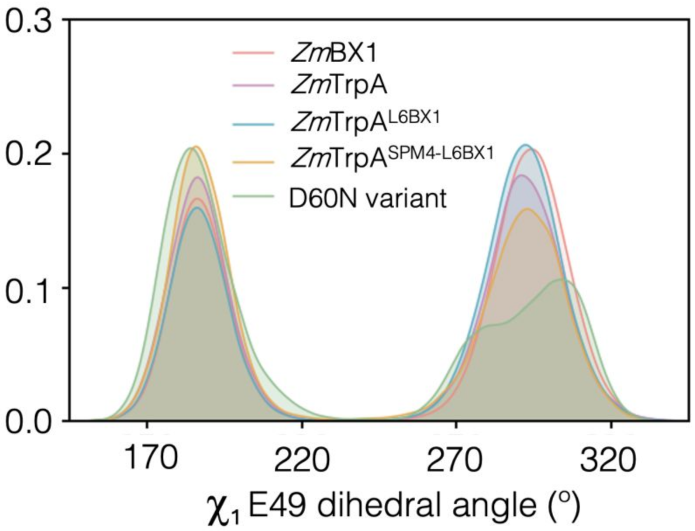

Figure S18. Histogram of the χ_1 dihedral of the catalytic E49 for all analyzed systems in the IGP-bound state. *Zm*BX1 is shown in pink, isolated *Zm*TrpA in purple, isolated *Zm*TrpA^{L6BX1} in blue, *Zm*TrpA$^{SPM4-L6BX1}$ in orange, and the D60N variant in green. Active states of E49 present χ_1 of ca. 185º, whereas inactive states values of 290º.

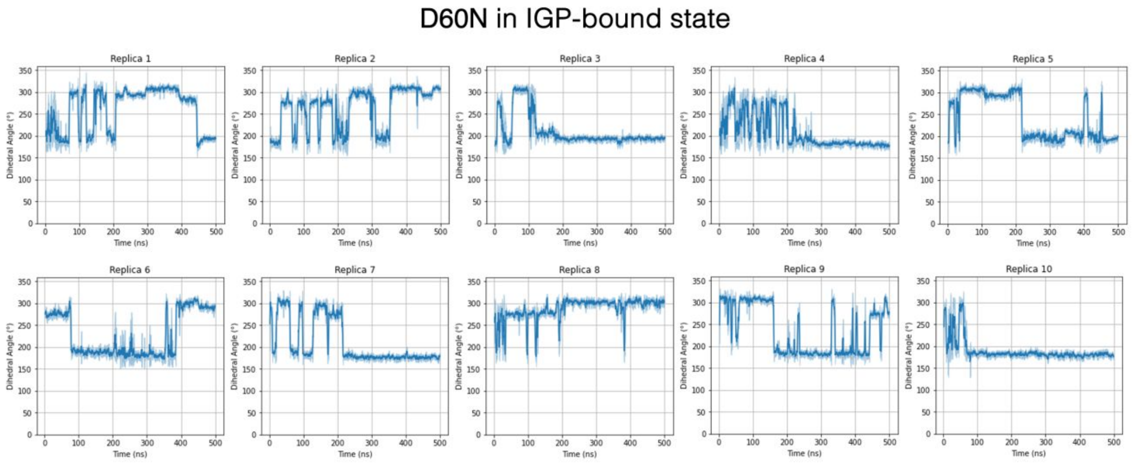

Figure S19. Plot of the E49 dihedral along the 500 ns MD simulation time for each replica and monomer of D60N variant in the IGP-bound state. The dihedral is in degrees.

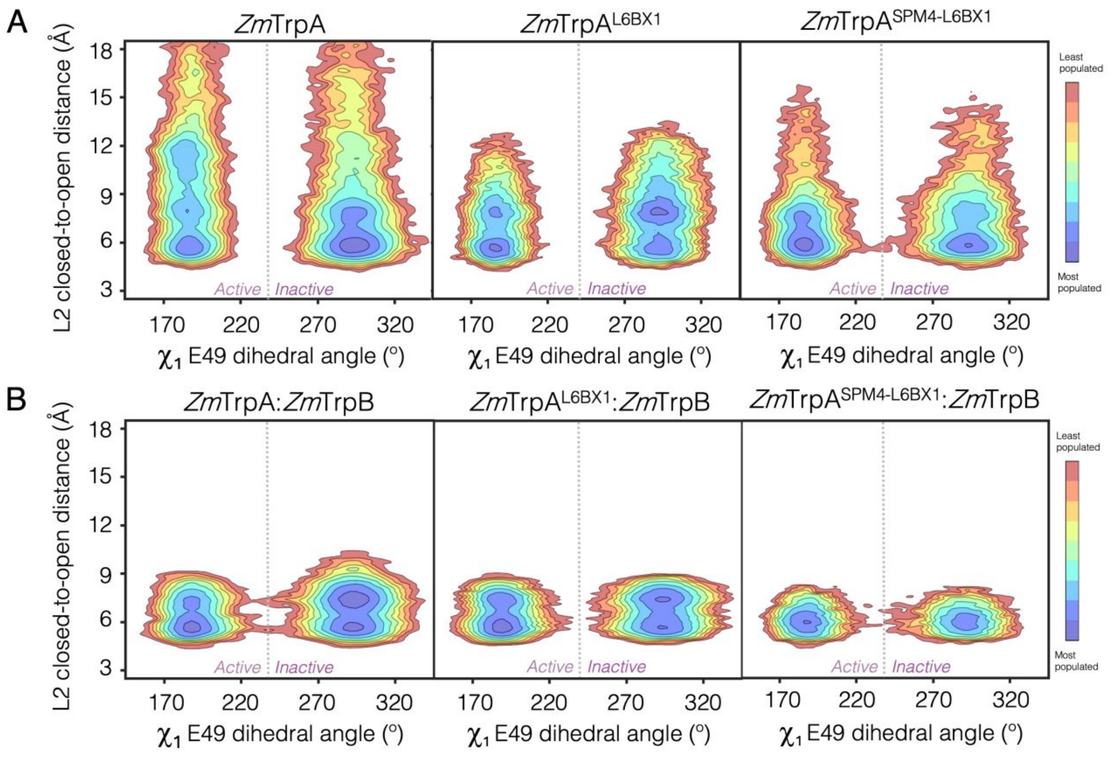

Figure S20. Conformational landscapes of several IGP-bound *Zm*TrpAs in the absence (A) and presence of TrpB (B). The reconstructed conformational landscapes are based on the dihedral χ_1 of the catalytic E49 (x-axis) and the L2 closed-to-open distances computed considering the distance between the carbon alpha of Y58 and

D125 (y-axis, in Å). Active states of E49 present χ_1 of ca. 185º, whereas inactive states values of 290º. Closed states of L2 (L2^C) present distances of ca. 5-6 Å, whereas L2 open (L2^O) and widely open (L2^{WO}) states distances of ca. 7 and 12 Å, respectively. Most stable conformations are colored in blue, whereas least stable ones in red.

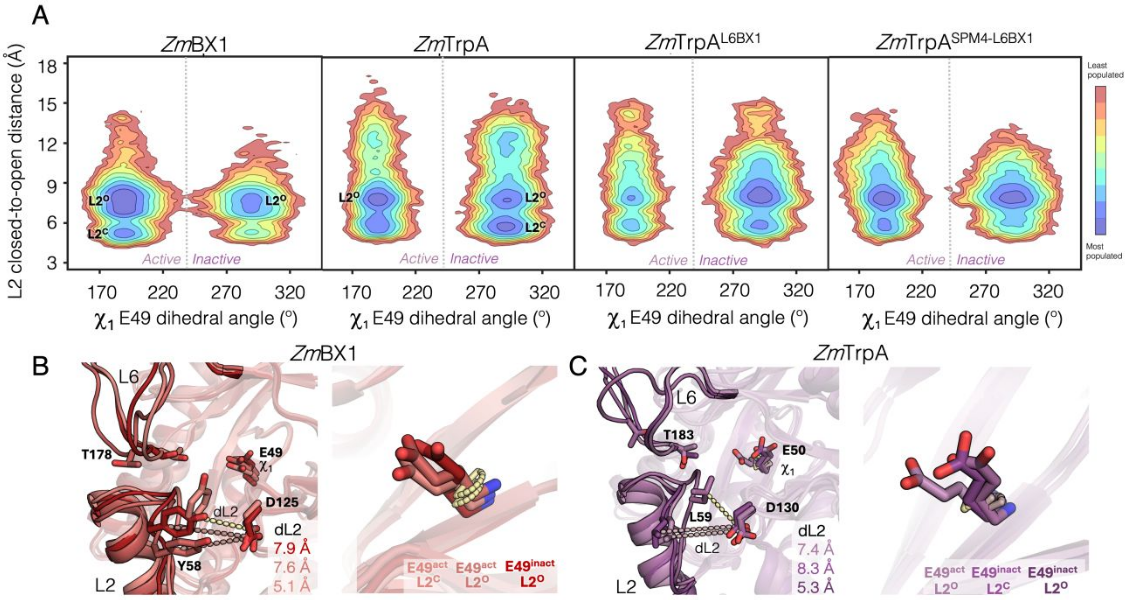

Figure S21. Conformational landscapes of several *Zm*TrpAs in the absence of IGP and binding partner. A. The reconstructed conformational landscapes are based on the dihedral χ_1 of the catalytic E49 (x-axis) and the L2 closed-to-open distances computed considering the distance between the carbon alpha of Y58 and D125 (y-axis, in Å). Active states of E49 present χ_1 of ca. 185º, whereas inactive states values of 290º. Closed states of L2 (L2^C) present distances of ca. 5-6 Å, whereas L2 open (L2^O) and widely open (L2^{WO}) states distances of ca. 7 and 12 Å, respectively. Most stable conformations are colored in blue, whereas least stable ones in red. Overlay of representative structures of the active and inactive states presenting L2 in either closed or open conformations for *Zm*BX1 (**B**) and *Zm*TrpA (**C**). MD simulations of *Zm*TrpA in the apo state are performed in the absence of *Zm*TrpB binding partner.

Table S4. Summary of the predicted E49 and D60 pka values for *Zm*BX1 and *Zm*TrpA systems, considering both E49^{act} and E49^{inact} conformations. Structures were extracted from IGP-state MD simulations, presenting short catalytic distances (< 4 Å). Notice that *Zm*TrpA is hardly sampling this short catalytically competent distance (see Figure 2). All pka values have been calculated at pH=7 using the APBS biomolecular solvation suite.[31]

		**pka E49**	**pka D60**
***Zm*BX1**	E49^{act}	8.75	4.74
	E49^{inact}	8.06	4.81
***Zm*TrpA**	E49^{act}	8.47	4.69
	E49^{inact}	8.66	5.36

References

(1) Jumper, J.; Evans, R.; Pritzel, A.; Green, T.; Figurnov, M.; Ronneberger, O.; Tunyasuvunakool, K.; Bates, R.; Žídek, A.; Potapenko, A.; et al. Highly accurate protein structure prediction with AlphaFold. *Nature* **2021**, *596* (7873), 583-589.
(2) Ester, M.; Kriegel, H.-P.; Sander, J.; Xu, X. A Density-Based Algorithm for Discovering Clusters in Large Spatial Databases with Noise. In Proc. of 2nd International Conference on Knowledge Discovery and, 1996.
(3) Jukič, M.; Konc, J.; Gobec, S.; Janežič, D. Identification of Conserved Water Sites in Protein Structures for Drug Design. *J Chem Inf Model* **2017**, *57* (12), 3094-3103.
(4) Pedregosa, F.; Varoquaux, G.; Gramfort, A.; Michel, V.; Thirion, B.; Grisel, O.; Blondel, M.; Prettenhofer, P.; Weiss, R.; Dubourg, V.; et al. Scikit-learn: Machine Learning in {P}ython. *J. Mach. Learn. Res.* **2011**, *12*, 2825-2830.
(5) *AMBER 2020*; University of California, San Francisco, 2020. (accessed.
(6) Wang, J.; Wolf, R. M.; Caldwell, J. W.; Kollman, P. A.; Case, D. A. Development and testing of a general amber force field. *J. Comp. Chem.* **2004**, *25* (9), 1157-1174.
(7) Schutz, C. N.; Warshel, A. What are the dielectric “constants” of proteins and how to validate electrostatic models? *Proteins* **2001**, *44* (4), 400-417.
(8) Bayly, C. I.; Cieplak, P.; Cornell, W.; Kollman, P. A. A well-behaved electrostatic potential based method using charge restraints for deriving atomic charges: the RESP model. *J. Phys. Chem.* **1993**, *97* (40), 10269-10280.
(9) Singh, U. C.; Kollman, P. A. An approach to computing electrostatic charges for molecules. *J. Comp. Chem.* **1984**, *5* (2), 129-145.
(10) *Gaussian 16 Rev. C.01*; Wallingford, CT, 2016. (accessed.
(11) Olsson, M. H. M.; Søndergaard, C. R.; Rostkowski, M.; Jensen, J. H. PROPKA3: Consistent Treatment of Internal and Surface Residues in Empirical pKa Predictions. *J. Chem. Theory Comput.* **2011**, *7* (2), 525-537. . Søndergaard, C. R.; Olsson, M. H. M.; Rostkowski, M.; Jensen, J. H. Improved Treatment of Ligands and Coupling Effects in Empirical Calculation and Rationalization of pKa Values. *J. Chem. Theory Comput.* **2011**, *7* (7), 2284-2295.
(12) Dunn, M. F. Allosteric regulation of substrate channeling and catalysis in the tryptophan synthase bienzyme complex. *Arch. Biochem. Biophys.* **2012**, *519* (2), 154-166.
(13) Tian, C.; Kasavajhala, K.; Belfon, K. A. A.; Raguette, L.; Huang, H.; Migues, A. N.; Bickel, J.; Wang, Y.; Pincay, J.; Wu, Q.; et al. ff19SB: Amino-Acid-Specific Protein Backbone Parameters Trained against Quantum Mechanics Energy Surfaces in Solution. *J. Chem. Theory Comput.* **2020**, *16* (1), 528-552.
(14) Roe, D. R.; Brooks, B. R. A protocol for preparing explicitly solvated systems for stable molecular dynamics simulations. *J. Chem. Phys.* **2020**, *153* (5), 054123.
(15) Darden, T.; York, D.; Pedersen, L. Particle mesh Ewald: An N·log(N) method for Ewald sums in large systems. *J. Chem. Phys.* **1993**, *98* (12), 10089-10092.
(16) McGibbon, Robert T.; Beauchamp, Kyle A.; Harrigan, Matthew P.; Klein, C.; Swails, Jason M.; Hernández, Carlos X.; Schwantes, Christian R.; Wang, L.-P.; Lane, Thomas J.; Pande, Vijay S. MDTraj: A Modern Open Library for the Analysis of Molecular Dynamics Trajectories. *Biophys J* **2015**, *109* (8), 1528-1532.

(17) Nguyen, H.; Roe, D. R.; Swails, J.; Case, D. A. PYTRAJ v1.0.0.dev1: Interactive data analysis for molecular dynamics simulations (v1.0.0.dev1). *Zenodo* **2016**.
(18) Roe, D. R.; Cheatham, T. E. PTRAJ and CPPTRAJ: Software for Processing and Analysis of Molecular Dynamics Trajectory Data. *J. Chem. Theory Comput.* **2013**, *9* (7), 3084-3095.
(19) Gowers, R. J.; Linke, M.; Barnoud, J.; Reddy, T. J. E.; Melo, M. N.; Seyler, S. L.; Domanski, J.; Dotson, D. L.; Buchouz, S.; Kenney, I. M.; et al. MDAnalysis: a Python package for the rapid analysis of molecular dynamics simulations. *Proc. of the 15th python in science conf.* **2016**, 98-105.
(20) Scherer, M. K.; Trendelkamp-Schroer, B.; Paul, F.; Pérez-Hernández, G.; Hoffmann, M.; Plattner, N.; Wehmeyer, C.; Prinz, J.-H.; Noé, F. PyEMMA 2: A Software Package for Estimation, Validation, and Analysis of Markov Models. *J. Chem. Theory Comput.* **2015**, *11* (11), 5525-5542.
(21) Becke, A. D. Density-functional thermochemistry. III. The role of exact exchange. *J. Chem. Phys.* **1993**, *98* (7), 5648-5652. . Lee, C.; Yang, W.; Parr, R. G. Development of the Colle-Salvetti correlation-energy formula into a functional of the electron density. *Phys. Rev. B* **1988**, *37* (2), 785-789.
(22) Marenich, A. V.; Cramer, C. J.; Truhlar, D. G. Universal Solvation Model Based on Solute Electron Density and on a Continuum Model of the Solvent Defined by the Bulk Dielectric Constant and Atomic Surface Tensions. *J. Phys. Chem. B* **2009**, *113* (18), 6378-6396.
(23) Chai, J.-D.; Head-Gordon, M. Long-range corrected hybrid density functionals with damped atom–atom dispersion corrections. *Phys. Chem. Chem. Phys.* **2008**, *10* (44), 6615-6620.
(24) Dapprich, S.; Komáromi, I.; Byun, K. S.; Morokuma, K.; Frisch, M. J. A new ONIOM implementation in Gaussian98. Part I. The calculation of energies, gradients, vibrational frequencies and electric field derivatives1Dedicated to Professor Keiji Morokuma in celebration of his 65th birthday.1. *J. Mol. Struct. THEOCHEM* **1999**, *461-462*, 1-21.
(25) S. Fernandes, H.; Ramos, M. J.; M. F. S. A. Cerqueira, N. molUP: A VMD plugin to handle QM and ONIOM calculations using the gaussian software. *J. Comp. Chem.* **2018**, *39* (19), 1344-1353.
(26) Maier, J. A.; Martinez, C.; Kasavajhala, K.; Wickstrom, L.; Hauser, K. E.; Simmerling, C. ff14SB: Improving the Accuracy of Protein Side Chain and Backbone Parameters from ff99SB. *J. Chem. Theory Comput.* **2015**, *11* (8), 3696-3713.
(27) Vreven, T.; Morokuma, K.; Farkas, Ö.; Schlegel, H. B.; Frisch, M. J. Geometry optimization with QM/MM, ONIOM, and other combined methods. I. Microiterations and constraints. *J. Comp. Chem.* **2003**, *24* (6), 760-769. . Vreven, T.; Byun, K. S.; Komáromi, I.; Dapprich, S.; Montgomery, J. A., Jr.; Morokuma, K.; Frisch, M. J. Combining Quantum Mechanics Methods with Molecular Mechanics Methods in ONIOM. *J. Chem. Theory Comput.* **2006**, *2* (3), 815-826.
(28) Okuta, R.; Unno, Y.; Nishino, D.; Hido, S.; Loomis, C. CuPy: A NumPy-Compatible Library for NVIDIA GPU Calculations. In *Conference on Neural Information Processing Systems (NIPS)*, 2017.
(29) Teixeira, C. S. S.; Ramos, M. J.; Sousa, S. F.; Cerqueira, N. M. F. S. A. Solving the Catalytic Mechanism of Tryptophan Synthase: an Emergent Drug Target in the Treatment of Tuberculosis. *ChemCatChem* **2020**, *12* (1), 227-237.

(30) Duran, C.; Kinateder, T.; Hiefinger, C.; Sterner, R.; Osuna, S. Altering Active-Site Loop Dynamics Enhances Standalone Activity of the Tryptophan Synthase Alpha Subunit. *ACS Catalysis* **2024**, *14* (22), 16986-16995. . Schupfner, M.; Busch, F.; Wysocki, V. H.; Sterner, R. Generation of a Stand-Alone Tryptophan Synthase α-Subunit by Mimicking an Evolutionary Blueprint. *ChemBioChem* **2019**, *20* (21), 2747-2751.
(31) Jurrus, E.; Engel, D.; Star, K.; Monson, K.; Brandi, J.; Felberg, L. E.; Brookes, D. H.; Wilson, L.; Chen, J.; Liles, K.; et al. Improvements to the APBS biomolecular solvation software suite. *Prot. Sci.* **2018**, *27* (1), 112-128.
